# In Vitro Assessment Methods for Antidiabetic Peptides from Legumes: A Review

**DOI:** 10.3390/foods12030631

**Published:** 2023-02-02

**Authors:** Alia Rahmi, Jayashree Arcot

**Affiliations:** Food and Health Group, School of Chemical Engineering, UNSW Sydney, Sydney, NSW 2052, Australia

**Keywords:** legume peptide, starch digestion inhibitor, glucose absorption inhibitor, diabetes management

## Abstract

Almost 65% of the human protein supply in the world originates from plants, with legumes being one of the highest contributors, comprising between 20 and 40% of the protein supply. Bioactive peptides from various food sources including legumes have been reported to show efficacy in modulating starch digestion and glucose absorption. This paper will provide a comprehensive review on recent in vitro studies that have been performed on leguminous antidiabetic peptides, focusing on the α-amylase inhibitor, α-glucosidase inhibitor, and dipeptidyl peptidase-IV (DPP-IV) inhibitor. Variations in legume cultivars and methods affect the release of peptides. Different methods have been used, such as in sample preparation, including fermentation (t, T), germination (t), and pre-cooking; in protein extraction, alkaline extraction, isoelectric precipitation, phosphate buffer extraction, and water extraction; in protein hydrolysis enzyme types and combination, enzyme substrate ratio, pH, and time; and in enzyme inhibitory assays, positive control type and concentration, inhibitor or peptide concentration, and the unit of inhibitory activity. The categorization of the relative scale of inhibitory activities among legume samples becomes difficult because of these method differences. Peptide sequences in samples were identified by means of HPLC/MS. Software and online tools were used in bioactivity prediction and computational modelling. The identification of the types and locations of chemical interactions between the inhibitor peptides and enzymes and the type of enzyme inhibition were achieved through computational modelling and enzyme kinetic studies.

## 1. Introduction

Diabetes is a global health problem and the world’s fastest growing chronic disease. Diabetes is one of the top ten causes of death in the world [1]. Over the course of 19 years (2000–2019), diabetes-related mortality increased by 3% [2]. Approximately 537 million adults worldwide between the ages of 20 and 79 years had diabetes mellitus in 2021. This number is estimated to increase to 643 million by 2030 and 783 million by 2045 [3].

There are three main types of diabetes: diabetes mellitus type 1 (T1DM), diabetes mellitus type 2 (T2DM), and gestational diabetes [3]. Among the three, T2DM is the most common, and affects 85–95% of the diabetic population worldwide [4].

T2DM is a chronic condition characterized by insulin deficiency and peripheral insulin resistance which causes high blood-glucose levels. Diets high in readily digestible carbohydrates (starch) and highly processed foods, as well as a sedentary life, play a crucial role in the epidemy of T2DM [5].

A healthy lifestyle is the foundation of T2DM management. This includes a healthy diet, regular physical exercise, not smoking, and maintaining a healthy body weight [3]. For some people with T2DM, practising a healthy lifestyle is difficult. For some others, over the long run of the course of illness, blood-glucose levels become difficult to maintain control of solely by following a healthy lifestyle. These difficulties cause T2DM patients to use hypoglycaemic drugs. Available oral hypoglycaemic drugs include metformin, sulfonylureas, rosiglitazone, acarbose, voglibose, miglitol, and gliptins. Oral hypoglycaemic drugs help control blood-glucose levels by reducing insulin resistance (metformin), stimulating insulin production (sulfonylureas), increasing insulin sensitivity (rosiglitazone), acting as an α-amylase inhibitor (acarbose), acting as α-glucosidase inhibitors (acarbose, voglibose, miglitol), and acting as a DPP-IV inhibitor (gliptins) [6,7,8,9,10,11,12,13,14]. Further, if treatment with oral hypoglycaemic medications no longer reflect in the desired blood-glucose level, people with T2DM may need insulin injections.

This paper focuses on in vitro studies that have been performed on leguminous antidiabetic peptides, i.e., an α-amylase inhibitor, an α-glucosidase inhibitor, and a DPP-IV inhibitor, in an attempt to understand the different methods used and their interpretations.

The inhibition of salivary and pancreatic α-amylases will cause some fractions of the starch and polysaccharides in the food bolus to remain undigested when reaching the small intestine. This will reduce the number of substrates for α-glucosidase. Further, the inhibition of α-glucosidase will cause the digested amylose and amylopectin of starch to remain as maltose, maltotriose, branching oligosaccharides, and isomaltose. These intermediate starch digesta fractions are hence not absorbable by intestinal cells for transfer into the blood stream. The inhibition of α-amylase and α-glucosidase will result in a decreased serum glucose level, which is beneficial in controlling the postprandial blood-glucose level [10,12].

**Figure 1:**
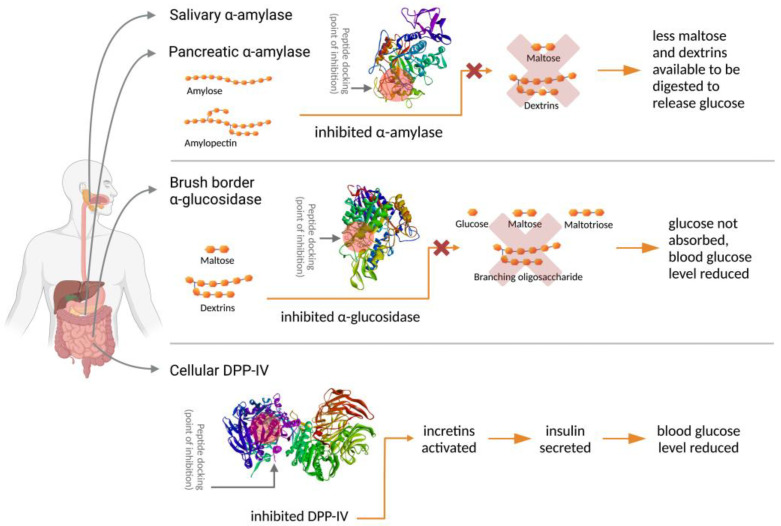
Inhibition mechanisms of α-amylase, α-glucosidase, and DPP-IV by peptides in controlling diabetes (Created with BioRender.com).

The inhibition of DPP-IV enzymes will prolong the biological activity of incretins. Incretins consist of two hormones, glucagon-like peptide 1 (GLP-1) and glucose-dependent insulinotropic peptide (GIP) [12,15]. These hormones are insulin secretagogues. When incretins are active, or when DPP-IV is inhibited, incretins enhance insulin secretion, which will keep the blood-glucose level under control. The inhibition mechanisms of α-amylase, α-glucosidase, and DPP-IV by peptides in controlling diabetes are presented in Figure 1.

Long-term use of hypoglycaemic synthetic drugs, however, can cause side effects such as bloating, diarrhoea, abdominal pain, fluid retention, osteoporosis, hypoglycaemia, improper function of the liver, risk of kidney injury, and heart failure [16,17]. This causes a demand for alternative treatments, which are safer while having a similar effect. As explained below, natural dietary compounds can offer viable options.

Researchers worldwide have attempted to investigate the use of natural dietary compounds to reduce starch digestibility and glucose absorption by means of increasing resistant starch, either through processing or through its addition into products [18]: the inclusion of soluble fibre [19]; the inclusion of lipids [20,21]; the inclusion of phenolic compounds [22]; the inclusion of proteins [23,24]; and the inclusion of protein hydrolysates and bioactive peptides [25,26,27]. The mechanisms by which these compounds act include: a reduction in the amount of digestible starch (resistant starch); an increased viscosity of the lumen fluid to hinder the binding between the starch-digestion enzymes and their substrate (soluble fibre and protein); a layering at the starch granule surface to hinder the binding between starch digestion enzymes and their substrate (lipid and protein); the inhibition of α-amylase, α-glucosidase, and dipeptidyl peptidase-IV/DPP-IV activity, the delay of glucose absorption in the small intestine, and a delay in glucose uptake by cells (phenolics, protein hydrolysates, bioactive peptides, and amino acids) [18,19,20,21,22,24,25,26,27,28].

The main objective of this review is to provide a comprehensive review of recent in vitro studies that have been performed on leguminous antidiabetic peptides, focusing on the α-amylase inhibitor, α-glucosidase inhibitor, and dipeptidyl peptidase-IV (DPP-IV) inhibitor in an attempt to understand the methods used and their interpretations.

## 2. Starch to Blood Glucose

**Figure 2:**
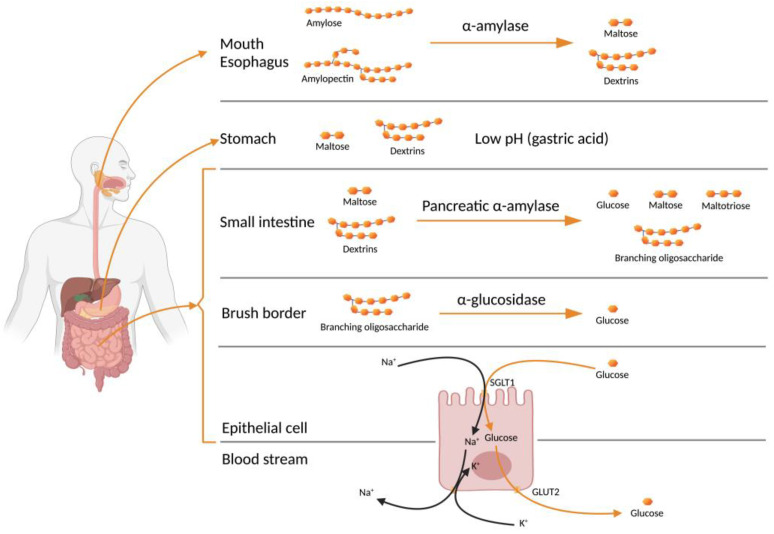
Starch digestion (Created with BioRender.com).

Starch (polysaccharide) digestion begins in the mouth, where α-1,4 glycosidic linkages are cleaved by salivary α-amylase. Figure 2 shows the starch digestion process, from the salivary α-amylase to its absorption into the blood stream as glucose. Digestion by α-amylase in the oral cavity will result in small amounts of monosaccharides as the food is rapidly swallowed and transferred to the stomach. The α-amylase activity continues after food is swallowed down into the stomach. It persists until the gastric acid penetrates the food bolus, and is then deactivated by the low-pH conditions. Dextrins and maltose predominate the digesta as it enters the small intestine. Pancreatic juice creates a neutral-pH buffering; this allows digestion to resume. The presence of α-amylase in the pancreatic juice hydrolyses the amylose chains into glucose, maltose and maltotriose [29]. The amylopectin chains are hydrolysed into glucose, maltose, branching oligosaccharides, and isomaltose. Both branching oligosaccharides and isomaltose have α-1,6 glycosidic linkages. Disaccharides such as α-glucosidase are further digested by enzymes at the brush borders (microvilli of mucosal cells); α-glucosidase is an important, membrane-bound enzyme in the epithelium of the small intestine that produces glucose [30]. The inhibition of α-amylase and α-glucosidase will result in a reduction in the number of glucose units available for absorption [9].

Glucose is absorbed through the intestinal lining cells or enterocytes, across the epithelial cell and into the blood stream inside the villus. Glucose enters the epithelium through the apical membrane and leaves it through the basolateral membrane. A sodium-dependent hexose transporter (SGLT1) carries glucose into the enterocyte by first orienting towards the intestinal lumen to bind with sodium. This binding causes the transporter to change its conformation and opens the pocket for glucose to bind. When glucose binds, the transporter reorients and moves glucose and sodium into the cell. The transporter releases sodium and glucose into the cytoplasm and returns to its lumen-facing orientation to pick up more glucose and sodium. The sodium level in the cytoplasm is maintained in balance with potassium by a battery of sodium–potassium pumps on the basolateral membrane. These pumps shuttle out sodium in exchange for potassium. Glucose is transferred into capillary blood in the villus through a facilitated diffusion by another hexose transporter (GLUT2) on the basolateral membrane [31].

The glucose concentration in the blood after a meal is called postprandial glucose. The glycaemic index (GI) is measured by the blood-glucose concentration after a meal. GI is a ranking of the carbohydrates in foods on a scale of 1–100, based on the extent to which they increase blood sugar levels after consumption. The GI is used to classify different sources of carbohydrate-rich foods by their effect on postprandial glycemia [32].

The normal concentration of circulating blood glucose is approximately 3.9–5.4 mmol/L (70–90 mg/dL). It is predominantly controlled by the two pancreatic hormones: insulin and glucagon. These are peptide hormones that lower and raise the blood-glucose concentration, respectively. Insulin stimulates glucose uptake by cells when the blood-glucose level is too high, while glucagon stimulates the conversion of stored glycogen in the liver to glucose when the blood-glucose level is too low. Secretions of both insulin and glucagon are regulated by another hormone, called an incretin. Incretins are a group of metabolic hormones that stimulate the decrease in blood glucose by the secretion of insulin and inhibit the release of glucagon. Incretins can be deactivated by the presence of dipeptidyl peptidase IV (DPP-IV), which can lead to an increase in the blood-glucose level through the secretion of glucagon and the inhibition of insulin release [33]. A DPP-IV inhibitor, used to inhibit the enzymatic activity of DPP-IV, can be used to treat T2DM to maintain the activity of incretins so that a decrease in the blood-glucose level is stimulated. 

## 3. Antidiabetic Activity of Leguminous Protein Hydrolysates and Peptides

Proteins are polymers of amino acids that are linked together by peptide bonds. There are hundreds of amino acids found in nature, but only 20 are found in the human body and other living organisms [34]. Through various combinations of sequence, amino acids are the building blocks of protein. Protein can be hydrolysed by hydrolytic enzymes in the gastrointestinal tract. Hydrolysis cleaves the peptide bonds in protein, and the long protein chains become shorter units called protein hydrolysates. Upon further hydrolysis, these protein hydrolysates are broken down into even smaller units called peptides and amino acids [35].

Short-chain amino acid sequences obtained from protein may have potential physiological functions in addition to their nutritional contribution [36]. The peptides, depending on the original protein–amino acid composition and sequence [37], may have beneficial physiological effects, such as being antihypertensive, acting as immunomodulators and antimicrobials, and having antidiabetic properties [38,39,40].

Specific protein fragments, i.e., peptides, that promote physiological functions and the conditions of human health are defined as bioactive peptides. In the sequence of a parent protein, bioactive peptides are inactive. They can be released through hydrolysis. Enzymatic hydrolysis is the main process for obtaining peptides from their parent proteins and allowing them to actively function biologically [37].

Bioactive peptides have been reported to show efficacy in modulating starch digestion and glucose absorption, such as the inhibition of α-amylase, α-glucosidase, and dipeptidyl peptidase IV (DPP-IV); the stimulation of insulin secretion; the decrease in glucose absorption in the gut; and the improvement of glucose uptake in the peripheral tissues [7,41,42,43,44]. One study found that peptides from the cowpea plant (*Vigna unguiculata* genotype Epace 10) have a similar amino acid sequence to that of bovine insulin [45]. The amino acid sequence of the bovine insulin α-chain is GIVEQCCASVCSLYQLENYCN, and protein isolated from *Vigna unguiculata* has the amino acid sequence GIVEQXXASVXSLYQLENYXN. The β-chain of the bovine insulin peptide sequence is FVNQHLCGSHLVEALYLVCGERGFFYTPKA, and protein isolated from *Vigna unguiculata* has the amino acid sequence FVNQHLXGSHLVEALYLVXGERGFFYTPKA. The X residue in the sequence is probably cysteine. Cysteine residues are not detected because the proteins are not reduced/alkylated [45]. Similarly, one review argued that cowpea peptides exhibited insulin-like properties [46]. They reached this conclusion based on the detection of these peptides in cowpea by Western blotting, and confirmed their role as immunoreactive insulin against anti-human insulin antibodies by an ELISA assay.

Dietary proteins, their protein hydrolysates, and their derived peptides from various food sources are vastly studied for their potential as therapeutic agents and have been proposed as alternative treatments for diabetes prevention and control. Comprehensive reviews on antidiabetic bioactive peptides from various food sources have been published [41,43,47,48,49,50,51]. In general, food-derived antidiabetic hydrolysates and peptides are isolated from two main sources, i.e., animal (dairy, egg, and marine) and plant (cereals and pseudocereals, rice, brewers’ spent grain, amaranth, quinoa, legumes, soybean, fruits, and leafy vegetables) [47].

Nearly 65% of the human protein supply in the world originates from plants [52], with legumes being one of the highest contributors to the protein supply, comprising 20–40% [36]. Legumes contain good-quality protein and are an economical dietary source of protein due to their high protein content, agronomical adaptability, economic value, and availability around the world. Therefore, in countries where animal protein sources are less accessible, legumes are widely consumed as the main source of dietary protein. Legume consumption as the main source of dietary protein indicates that legumes potentially have no side effects [53] and are also potentially safe when consumed as therapeutic agents, such as bioactive peptides. Cytotoxicity assays on cowpea protein hydrolysates and peptide fractions performed against Vero Cells showed very low cytotoxic effects when compared to the control, docetaxel (Taxotere^®^), even under maximum concentrations of 0.45–0.9 mg protein/mL [54]. Therefore, cowpea protein hydrolysates and peptide fractions are potentially safe. Moreover, peptides are reported to present low toxicity levels and low accumulation in body tissues [36].

Several reports claim that legume consumption is associated with a reduction in the risk of diabetes [13,55,56]. Studies have shown that this property is attributed to the presence of resistant starch, fibre, polyphenols, and bioactive peptides, which are released naturally in the stomach and intestine during digestion [57].

Until now, information on leguminous antidiabetic peptides, i.e., α-amylase, α-glucosidase, and DPP-IV inhibitors, have been limited. Leguminous peptide research is currently focused on other bioactivities, including antioxidant and antihypertension effects. On the other hand, the research on antidiabetic peptides has been using animal and other plant protein sources. This paper will provide a comprehensive review on in vitro studies that have been performed on leguminous antidiabetic peptides in an attempt to understand the different methods used and their interpretations.

Based on their antidiabetic activity analysis, leguminous antidiabetic peptide studies can be divided into four categories: in vitro, cell work, in vivo and ex vivo, and in silico. This paper will cover only the in vitro studies of leguminous antidiabetic peptides obtained through enzymatic hydrolysis. The in vitro studies are listed in Table 1.

## 4. In Vitro Studies on Leguminous Antidiabetic Peptides

Several common bean (*Phaseolus vulgaris* L.) cultivars and preparation techniques have been investigated for their antidiabetic peptides using in vitro assays. Soybeans (*Glycine max*), cowpeas (*Vigna unguiculata*), and bambara beans (*Vigna subterranean*) have also been studied. Common beans, soybeans, cowpeas, and bambara beans which have been reported for their α-amylase, α-glucosidase, and DPP-IV inhibitory peptides through in vitro assays are shown in Table 1.

Research on leguminous antidiabetic peptides using in vitro assays generally involves sample preparation, protein extraction/fractionation, protein hydrolysis, inhibitory assays, molecular-mass profiling, peptide sequence identification, potential bioactivity prediction of the peptide, computational modelling, and enzyme kinetics studies. Some studies include the synthesis of selected peptides to reconfirm their inhibitory activities. Similar to this general workflow, Nongonierma and Fitzgerald illustrated the same conventional approach in their research on biologically active peptides derived from food-protein [58].

**Table 1:** In vitro studies on antidiabetic leguminous protein hydrolysates and peptides.

Legumes	Sample Type	Highest α-Amylase Inhibition *		Highest α-Glucosidase Inhibition *		Highest DPP-IV Inhibition *		References
		Value	Sample	Value	Sample	Value	Sample	
Common Bean (*Phaseolus vulgaris* L.): Black Pinto Red Navy Great Northern	Raw and precooked	36% inh AC/mg protein [Prot] = not reported [Enz] = 13 U/mL [Ac] = 1 mM	Red beans Raw, whole H: Pepsin-Pancreatin	>40% (~48–67%) inh AC /mg protein (not statistically different) [Prot] = not reported [Enz] = 1 U/mL [Ac] = 1 mM	All beans Raw and precooked, whole H: Pepsin-Pancreatin	0.093 mg protein/mL [Prot] = 1 mg DW/mL [Enz] = 100 ng/mL [control] = not reported	Navy beans Precooked, whole H: Pepsin-Pancreatin	[59]
						0.095 mg protein/mL [Prot] = 1 mg DW/mL [Enz] = 100 ng/mL [control] = not reported	Navy beans Raw, whole H: Pepsin-Pancreatin	
Common Bean (*Phaseolus vulgaris* L.): Mexico, Pinto: Pinto-Bayacora Pinto-Bravo Pinto-Centenario Pinto-Saltillo Mexico, Flores de Mayo and Junio: FMayo-Eugenia FMayo-67 FMayo-199 FMayo-202 FJunio-Leon FJunio-Marcela Mexico, Negros: Negro-Frijozac Negro-Otomi Brazil, Carioca: BRSHorizote BRS-Pontal Perola	Raw	14.9 ± 1.7% inh AC/mg BPI [Prot] = not reported [Enz] = 13 U/mL [Ac] = 1 mM	Pinto-Bayacora Raw, dehulled H: Pepsin-Pancreatin					[38]
		14.9 ± 0.4% inh rel AC/mg BPI [Prot] = not reported [Enz] = 13 U/mL [Ac] = 1 mM	FMayo-67 Raw, dehulled H: Pepsin-Pancreatin					
Common Bean (*Phaseolus vulgaris* L.): Black Otomi BRS-Horizonte BRS-Pontal Perola	Raw			50.10% inh/mg DW [Prot] = 1 mg DW/mL [Enz] = 1 U/mL [Ac] = 1 mmol/L	BRS-Horizonte Raw, dehulled H: Pepsin-Pancreatin	0.14 mg DW/mL [Prot] = 1 mg DW/mL [Enz] = 10 ng/mL [control] = not reported	Black Otomi Raw, dehulled H: Pepsin-Pancreatin	[60]
				49.34% inh/mg DW [Prot] = 1 mg DW/mL [Enz] = 1 U/mL [Ac] = 1 mmol/L	Synthesized peptide: KKSSG	0.03 mg DW/mL [Prot] = 1 mg DW/mL [Enz] = 10 ng/mL [control] = not reported	Synthesized peptide: KTYGL	
Black Otomi (*Phaseolus vulgaris* L.)	Raw	64.5 ± 2.7% inh/ mg dry matter [Prot] = 1 mg DM/mL [Enz] = 13 U/mL [Ac] = 1 mM	Raw, dehulled H: Flavourzyme, 2 h, 1:20 (E/S)	75.3 ± 0.7% to 78.4 ± 0.6% inh/mg dry matter (not statistically different) [Prot] = 1 mg DM/mL [Enz] = 1 U/mL [Ac] = 1 mmol/L	Raw, dehulled H: Papain, 2, 3, 4 h, 1:20, 1:30, 1:50 (E/S)	96.7% inh/ mg dry matter [Prot] = 1 mg DW/mL [Enz] = 10 ng/mL [control] = not reported	Raw, dehulled H: Alcalase, 2 h, 1:20 (E/S)	[61]
HTC Common Bean (*Phaseolus vulgaris* L.): cv Negro 8025 cv. Pinto Durango	Raw	49.9 ± 1.4% [Prot] = 100 μg/mL [Enz] = 10.8 U/mL [Ac] = 1 mM	Pinto Durango Raw, dehulled H: Bromelain, 2 h F: <1 kDa	76.4 ± 0.5% [Prot] = not reported [Enz] = 1.0 U/mL [Ac] = 1 mM	Pinto Durango Raw, dehulled H: Alcalase, 2 h F: <1 kDa	55.3 ± 1.6% [Prot] = 100 μg/mL [Enz] = 100 ng/mL [control] = not reported	Pinto Durango Raw, dehulled H: Alcalase, 2 h F: <1 kDa	[25]
Common Bean (*Phaseolus vulgaris*)	Germinated	30.88 ± 2.45% AC/mg SP [Prot] = 1 mg/mL [Enz] = 13 U/mL [Ac] = 1 mM	Dehulled, germinated 24 h H: Non-hydrolyzed			1.2 mg soluble protein/mL [Prot] = 0.1–4.0 mg/mL [Enz] = 100 ng/mL [control] = not reported	Dehulled, non-germinated H: Non-hydrolyzed	[55]
Pinto Bean (*Phaseolus vulgaris* cv. Pinto)	Raw	57.48 ± 2.51% [Prot] = not reported [Enz] = 0.5 mg/mL [Ac] = not used	Raw, whole H: Protamex, pH 6.5, 1 h, 1:10 (E/S)					[42]
		62.1 ± 3.49% [Prot] = not reported [Enz] = 0.5 mg/mL [Ac] = not used	Raw, whole H: Protamex, pH 6.5, 1 h, 1:10 (E/S) F: <3 kDa					
Pinto Bean (*Phaseolus vulgaris* cv. Pinto)	Raw	57.8% inh/100 μg 10.03 ± 0.47 mM [Prot] = 1 mg/mL [Enz] = 0.5 mg/mL [Ac] = not used	Raw, whole H: Protamex, pH 6.5, 1 h, 1:10 (E/S) F: <3 kDa Synthesized pinto bean peptide fraction 5 (PBp5)					[62]
Bean (*Phaseolus vulgaris* L.)	Fermented with *L. plantarum 299v*	0.038 µg/mL [Prot] = not reported [Enz] = not reported [Ac] = not used	Dehulled Fermented 22 °C, 3 h H: Amylase-Pepsin-Pancreatin F: Fraction III collected in Sephadex G-10 from 3.5–7 kDa					[63]
BRS-Pontal (*Phaseolus vulgaris* L.)	Raw	89.1 ± 0.3% [Prot] = 10 mg/mL [Enz] = 10 U/mL [Ac] = 10 mg/mL	Hard-to-cook bean, raw, dehulled H: Non-hydrolyzed F: <3 kDa	89.2 ± 0.1% [Prot] = 10 mg protein/mL [Enz] = 2 U/mL [Ac] = 10 mg/mL	Easy-to-cook bean, raw, dehulled Non-hydrolyzed F: <3 kDa			[7]
Carioca Bean (*Phaseolus vulgaris* L. cv Carioca)	Raw	101.61 ± 0.78% [Prot] = 1 mg/mL [Enz] = not reported [Ac] = not used	Raw, whole H: Alcalase-Neutrase (1/2:1/2)	34.73 ± 4.65% [Prot] = 1 mg/mL [Enz] = 0.1 U/mL [Ac] = not used	Raw, whole H: Flavourzyme: Alcalase (1/2:1/2)			[64]
Cowpea cultivar BRS Novaera (*Vigna unguiculata* L.)	Germinated					0.58 mg soluble protein/mL [Prot] = 0.1–4.0 mg/mL [Enz] = 100 ng/mL [control] = not reported	Dehulled, Non-germinated H: Alcalase, 1 h-Pepsin-Pancreatin	[57]
Black Cowpea (*Vigna unguiculata)*	Raw	96.81% [Prot] = 100 mg/mL [Enz] = 13 U/mL [Ac] = not reported	Raw, whole H: Pepsin-Pancreatin F: <1 kDa	97.34% [Prot] = 10 mg/mL [Enz] = 2 U/mL [Ac] = not reported	Raw, whole H: Alcalase-Flavourzyme F: >10 kDa	85% 2.06 mg protein/mL [Prot] = not reported [Enz] = not reported [Stg] = not reported	Raw, whole H: Alcalase-Flavourzyme F: Protein Hydrolysate	[54]
Bambara bean (*Vigna subterranea*)	Raw					44.253 ± 1.327% [Prot] = 1 mg/mL [Enz] = 0.26 mU/test well [DipA] = not reported	Bambara bean protein isolate H: Alcalase	[13]
						29.276 ± 0.878% at 1 mg/mL [Prot] = 1 mg/mL [Enz] = 0.26 mU/test well [DipA] = not reported	Bambara bean protein isolate H: Trypsin-Pepsin-α-chymotrypsin-trypsin-pancreatin	
						1.733 mg/mL [Prot] = 1 mg/mL [Enz] = 0.26 mU/test well [DipA] = not reported	Bambara bean protein isolate H: Alcalase H: Thermolysin	
Soybean (*Glycine max*)	Germinated	1.7 mg/mL [Prot] = 0.2–4 mg/mL [Enz] = 2 U/mL [Ac] = 0.1–1.3 mg/mL	Germinated 6 days, whole H: Pepsin-Pancreatin	As maltase 2.56 mg/mL [Prot] = 1–10 mg/mL [Enz] = 1 U/mL [Ac] = not used	Germinated 6 days, whole H: Pepsin-Pancreatin F: <5 kDa	0.91 mg/mL [Prot] = 0.08–5 mg/mL [Enz] = 0.26 mU/test well [DipA] = 0.78–50 μM	Germinated 6 days, whole H: Pepsin-Pancreatin F: 5–10 kDa	[16]
				As sucrase 1.23 mg/mL [Prot] = 1–10 mg/mL [Enz] = 1 U/mL [Ac] = not used	Germinated 6 days, H: Pepsin-Pancreatin F: <5 kDa			
		~85% [Prot] = 1 mg/mL [Enz] = 2 U/mL [Ac] = 0.1–1.3 mg/mL	Germinated 6 days, whole H: Pepsin-Pancreatin F1 fraction collected by semi-preparative RP-HPLC from 5–10 kDa	As maltase 32% [Prot] = 1 mg/mL [Enz] = 1 U/mL [Ac] = not used	Germinated 6 days, whole H: Pepsin-Pancreatin F: F4 fraction collected in semi-preparative RP-HPLC from 5–10 kDa	0.7 mg/mL [Prot] = 0.08–5 mg/mL [Enz] = 0.26 mU/test well [DipA] = 0.78–50 μM	Germinated 6 days, whole H: Pepsin-Pancreatin F: F3 fraction collected by semi-preparative RP-HPLC from 5–10 kDa	
				As sucrase 22% [Prot] = 1 mg/mL [Enz] = 1 U/mL [Ac] = not used	Germinated 6 days, whole H: Pepsin-Pancreatin F: F1 fraction collected by semi-preparative RP-HPLC from 5–10 kDa			
Soybean	Raw (Isolated soybean protein)			0.27 mg/mL [Prot] = not reported [Enz] = 0.15 U/mL [Ac] = not used	Protein isolate H: Trypsin F: <5 kDa			[65]
				0.049 mg/mL [Prot] = not reported [Enz] = 0.15 U/mL [Ac] = not used	Protein isolate H: Trypsin F: <5 kDa Fraction C-III-2a from RP-HPLC			
Soy	Raw (Soy protein powder)			77.64 ± 1.07% [Prot] = not reported [Enz] = 0.2 U/mL [Ac] = 10 mg/mL	Soy protein isolate H: Alkaline protease-pepsin-pancreatin	47.94 ± 1.10% [Prot] = not reported [Enz] = 0.02 U/mL [control] = buffer	Soy protein isolate H: Alkaline protease-pepsin-pancreatin	[12]
				87.10 ± 2.70% [Prot] = not reported [Enz] = 0.2 U/mL [Ac] = 10 mg/mL	Soy protein isolate H: Alkaline protease-pepsin-pancreatin F: H1 fraction from DEAE-52			
				95.35 ± 2.70% [Prot] = not reported [Enz] = 0.2 U/mL [Ac] = 10 mg/mL	Soy protein isolate H: Alkaline protease-pepsin-pancreatin F: H1 fraction from DEAE-52, then H1-2 fraction from Sephadex G-15			
				162.29 ± 0.74 μmol/L [Prot] = not reported [Enz] = 0.2 U/mL [Ac] = 10 mg/mL	Synthesized peptide: WLRL			
Yellow field pea (*Pisum sativum* L.)	Raw (Yellow field pea protein concentrate)	30.52 ± 0.01% [Prot] = 225 μg/mL [Enz] = 28.57 μg/mL [Ac] = 1.5–3 μg/mL	Yellow field pea protein concentrate H: Chymotrypsin F: 1–3 kDa	53.35 ± 2.78% [Prot] = 20 mg/mL [Enz] = 8.33 mg/mL [Ac] = 0.00625–0.125 mg/mL	Yellow field pea protein concentrate H: Chymotrypsin F: <1 kDa			[9]

* Units in % indicate the percent inhibition; units in inh AC—relative to acarbose; mg (protein; DW; hydrolysate; soluble protein)/mL, µg/mL, or μmol/L indicate the IC50; BPI—bean protein isolate; SP—soluble protein; Prot—protein, peptide, hydrolysate, inhibitor; Enz—α-amylase, α-glucosidase, DPP-IV; Ac—acarbose; Stg—sitagliptin; DipA—diprotin A; H—hydrolysate; F—hydrolysate fraction.

Several studies have demonstrated factors that affect the release of peptides. These factors have been controlled in studies to show their effects, such as cultivars, sample preparation, hydrolysis optimisation, peptide fractionation, and semi-preparative peptides. Common beans from various cultivars release different peptide sequences that exhibit antidiabetic activities. Sample preparations such as fermentation (t, T), germination (t), and pre-cooking also affect the release of peptides and their antidiabetic activities. By designing experiments on enzyme types and combinations, enzyme substrate ratios (ESR), pH, and time, hydrolysis optimisations using the response surface methodology (RSM) have shown that the length and bioactivity of peptides released are affected. Peptide fractionations by means of ultrafiltration membranes from 1 to 100 kDa, ion-exchange chromatography, gel filtration, or reversed, phase-high performance liquid chromatography (RP-HPLC) were used in the studies. Semi-preparative peptides were obtained using solid-phase methods and standard 9-fluorenylmethyloxycarbonyl (Fmoc) chemistry. The methods used in the studies reflect the influence of these factors. They are discussed in more detail in the following sections.

Sample preparation generally begins with pod separation, sanitisation, rinsing, soaking, germination, fermentation, milling, oven drying, lyophilisation, grinding, and sieving. Protein extraction is performed to isolate protein fractions and ensure no other inhibitory compounds, such as resistant starch, fibre, and phenolic compounds, are present in the samples. Alkaline extraction and the isoelectric precipitation method are commonly used for this purpose. Protein hydrolysis is the focus of several research studies involving optimisation by experimenting on the enzyme types, enzyme mix composition, ESR, pH, and time. Inhibitory assays target in vitro α-amylase, α-glucosidase, and DPP-IV. Samples that show high inhibitory activity is usually investigated further for its peptide’s molecular-mass profile, sequence, potential bioactivity, molecular docking, and enzyme kinetics. The most promising peptide is synthesized using 9-fluorenylmethyloxycarbonyl (Fmoc) chemistry.

Information from molecular-mass profiling will assist the succeeding step: peptide sequence identification. Molecular-mass profiling is mostly performed using sodium dodecyl sulphate polyacrylamide gel electrophoresis (SDS-PAGE) and matrix-assisted laser desorption/ionization-time of flight mass spectrometry (MALDI-TOF/TOF MS). Peptide sequence identification makes use of high-performance liquid chromatography/mass spectrometry (HPLC/MS). Online peptide database resources such as BLAST^®^ Tool, PepDraw Tool, and the BIOPEP database are commonly used to confirm the peptide sequence, to predict the peptide structure and physicochemical properties, and to predict the peptide bioactivity, respectively.

Lastly, when used together, computational modelling and an enzyme kinetics study can predict the inhibition mechanism. Information about peptide molecular docking onto the enzymes, such as the types and locations of chemical interactions and the type of inhibition (such as competitive or non-competitive inhibitions), can be investigated using molecular docking software and Lineweaver–Burk plots.

Research that focused on investigating peptides for production as an alternative to the currently available antidiabetic drugs, such as the studies by Jiang et al. in 2018 [65] and Ngoh and Gan in the same year [62], took further steps to synthesise the most potent peptides. The synthesised peptides wee then subjected again to enzyme inhibitory assays for the reconfirmation of activity. Peptide synthesis is usually performed by biotechnology companies using Fmoc chemistry. Fmoc is a chemical synthesis method that produces peptides using an automated solid phase. In this method, a base-sensitive Fmoc (9-fluorenylmethyloxycarbonyl) group or an acid-sensitive Boc (*tert*-butoxycarbonyl) group are typically used as the α-amino-protecting group. Both the α-amino group and the side-chain protection are immobilized to a resin [66]. Other than chemical synthesis, recombinant DNA (rDNA) technology has been used to express proteins for structural studies [67], but there is no report indicating the use of this method in leguminous antidiabetic peptides research.

Inhibitory activities of leguminous antidiabetic peptides were reported in percent inhibition, or IC_50_. Acarbose was used by most studies as a positive control in α-amylase and α-glucosidase inhibitory assays. In DPP-IV inhibitory assays, diprotin A and sitagliptin were used as positive controls. The concentration of positive controls varied between studies. Acarbose concentration ranged between 1.5 μg/mL and 10 mg/mL in the α-amylase inhibitory assays and between 6.25 μg/mL and 10 mg/mL in the α-glucosidase inhibitory assays. Diprotin A was used between 0.78 and 50 μM. The majority of the studies used a DPP-IV Inhibitor Kit assay, which provides a pro-luminescent DPP-IV substrate (Gly-Pro-aminoluciferin or H-Gly-Pro-AMC) that is cleaved by DPP-IV to yield a fluorescent product (aminoluciferin or 7-amino-4-methylcoumarin). The fluorescent intensity was measured at 460 nm and was proportional to the DPP-IV activity. Protein or peptide samples containing the inhibitory peptides were prepared in various concentrations, ranging between 0.05 and 50 mg/mL in the α-amylase inhibitory assays, 1 and 200 mg/mL in the α-glucosidase inhibitory assays, and 0.08 and 5 mg/mL in the DPP-IV inhibitory assays [7,9,12,13,16,25,38,42,54,55,57,59,60,61,62,63,64,65]. Discussions on positive control concentrations and their interpretations are provided in their respective sections, i.e., α-amylase inhibitory assays, α-glucosidase inhibitory assays, and DPP-IV inhibitory assays.

The highest activity of α-amylase inhibition ranged between 14.9 and 89.1% relative to acarbose [7,9,16,25,38,55,59,61] and between 57.48 and 101.61% among assays that did not use acarbose [42,54,62,63,64]. IC_50_ ranged between 0.038 μg/mL and 1.7 mg protein/mL, and one study reported 10.03 mM. The highest activity of α-glucosidase inhibition ranged between 22 and 97.34% inhibition relative to acarbose [7,9,12,16,25,53,54,59,61], and was 34.73% in a non-acarbose assay [64]. IC_50_ ranged between 0.27 and 162.29 mg protein/mL. The highest activity of DPP-IV inhibition ranged between 47.94 and 96.7% inhibition and an IC_50_ between 0.03 and 1.2 mg/mL [12,13,16,25,53,54,55,57,59,61]. Discussions on factors affecting inhibitory activities are provided in their respective sections, i.e., α-amylase inhibitory assays, α-glucosidase inhibitory assays, and DPP-IV inhibitory assays.

## 5. Protein Extraction

Protein extraction is commonly performed using alkaline extraction and isoelectric precipitation methods. Other methods used include water extraction and phosphate buffer extraction. Protein extraction is a crucial step in isolating the protein fraction of the bean and minimising the presence of other potential inhibitors, such as resistant starch, fibres, and phenolic compounds. Some studies skip this step by using a commercially available protein isolate.

In general, prior to protein extraction, samples are prepared by separating the bean from the pod, sanitisation, rinsing, soaking, dehulling, germination, fermentation, blanching, precooking, milling, oven drying, lyophilisation, grinding, and sieving. Details are provided on Table 2. Milling, drying, and sieving are the basic steps in sample preparation. The use of freeze drying is very common. When a conventional oven is used, the drying temperature varies between 40 and 60 °C. The particle size of ready-to-extract samples varies between 20 and 60 mesh.

Dehulling is a crucial step in all studies. As bean proteins are mainly located in the cotyledons [74] and bean hulls contain fibres and phenolic compounds, the inclusion of hulls in the samples may increase the risk of the co-extraction of fibres and a phenolic load in the final protein isolate. Fibres and phenolic compounds have been proven to show potential antidiabetic properties by many studies. One study argued a higher inhibition potential due to synergistic inhibitory activity between the protein and phenolic compounds during digest because hulls were not removed from their sample [53]. The protein extraction method should be selected based on its specificity to extract the protein fractions. A study on soybeans using alkaline extraction without isoelectric precipitation and directly followed by protein hydrolysis resulted in a digesta containing total identified phenolics of 466.15 ± 21.20 μg/g [75]. In addition to than dehulling and careful method selection, it is also suggested to add an additional alcohol extraction step to remove any co-extracted phenolic compounds. Soy protein isolate can retain notable amounts of isoflavones associated with proteins [16,73]. Total phenolic-content determination can be performed to ensure the removal.

Where alkaline extraction and isoelectric precipitation are used, the ratios between the bean flour sample and water ranged between 1:5 and 1:20. The more water was used, the more protein could be extracted from the legumes. Most studies used a combination of pH 8.0 and 4.3 [7,25,38,53,55,57,59,61]. The pH selection is related to protein solubility (at basic pH) and isoelectric point (at acidic pH). Extraction was carried out at room temperature or 35 °C for 1 h with stirring. The centrifugation speed ranged between 4000× *g* and 10,000× *g* at 4, 20, and 25 °C. The effects of extraction temperature and centrifugation speed and temperature have not been discussed in studies in the field.

The protein extraction yield usually reflects the bean protein content; typically around 20%. Studies in the field did not report the protein yield data, except for Ngoh and Gan [42], who reported that protein extraction using a phosphate buffer generally yielded between 33.8 and 57.9 mg protein/g or around 3.38–5.79% from pinto flour [42]. The protein extraction yield from a cooked bean is significantly lower when performed at a pH < 10 [76]. A study on common beans, which included precooking, extracted the protein at a pH of 8 [59] and did not report the protein yield; however, theoretically, the yield should have been low.

The protein content of the isolates were reported between 68.11 and 90.51%, as follows: 68.11–80.17% [55], 71.7–78.7% [57], 78.6–79.7% [25], 86.62% [12], 90.26% [13], and 90.51% [64]. The protein-content determination was performed using the Kjeldahl method [12], the Lowry method [54,63,64], the Protein DC Microplate Assay of Bio-Rad [55,57,59], or the Qubit^®^ Protein Assay Kit [7]. The protein content in isolates is an important indicator of purity and will assist in determining the enzyme–substrate ratio for hydrolysis.

## 6. Protein Hydrolysis

Enzymatic hydrolysis is used in research on leguminous antidiabetic peptides to obtain protein hydrolysates and peptide fractions. Some studies focus on optimising this step by experimenting on enzyme types, enzyme mix composition, ESR (enzyme–substrate ratio), pH, and time. It can be highlighted that the hydrolysis method by Megías et al. [77] has been used in many studies. Detailed parameters used in protein hydrolysis are provided in Table 3.

Enzymes used were Alcalase, flavourzyme, protamex, trypsin, neutrase, α-chymotrypsin, bromelain, papain, thermolysin, protease K, alkaline protease, and α-amylase. The use of these enzymes was mostly followed by pepsin and pancreatin. The latter step was taken to simulate human gastro-intestinal digestion; this was a step taken in almost all the studies in the field [7,12,13,16,25,38,53,54,55,57,59,61,63]. Although it is not a protease, α-amylase was used in a study [63] to release sugar-bound proteins, which increases the protein availability to pepsin and pancreatin. Depending on the type of enzymes used in the enzymatic hydrolysis, different chain lengths of peptide sequences can be released, leading to a broad range of peptide bioactivities [51].

The protein hydrolysis yield was 44.03%, 48.18%, 49.56%, and 58.21% using trypsin, chymotrypsin, pepsin, and Alcalase, respectively [9]. The protein hydrolysis yield represents the percent ratio of total protein present in the final hydrolysate to the amount of total protein initially used in the hydrolysis. The protein content of the hydrolysates varied between studies, depending on the method of analysis. For example,0.43–0.78 mg/mL [54], 54.69–87.24% soluble protein [55], 63.5–73.9% of soluble protein [57], and 71.54–87.17% of freeze-dried hydrolysate [9]. The methods used in protein-content determination were the Kjeldahl and Lowry methods or the Protein DC Microplate Assay of Bio-Rad. The degree of hydrolysis was reported with a broad range: between 5.92 and 98.38%. Authors reported that the broad range was contributed to by variations in seed cultivar [9], sample preparation [63], protein extraction methods [9], nature of protein substrates [13], and the enzyme types and specificities [12,13]. The protein hydrolysis yield and the hydrolysate protein content, along with the degree of hydrolysis, can be used as practical references in designing the protein extraction and protein hydrolysis methods.

After hydrolysis, the protein hydrolysates were subjected to clarification, fractionation, separation, purification, and synthesis. Clarification was intended for salt removal using 0.5 kDa, 0.8 kDa, 3 kDa, or 0.45 μm of membrane. Fractionation was used to separate peptide fractions using an ultrafiltration membrane of 1, 3, 3.5, 5, 7, 10, 30, 50, or 100 kDa. Separation and purification were performed to isolate selected peptides that potentially have a high inhibitory activity. The peptides were isolated using ion-exchange chromatography, gel filtration, or RP-HPLC. Some of the studies synthesised the peptides that had the highest inhibitory activity [53,62]. Fmoc chemistry was reported as the method used in peptide synthesis [12,65].

## 7. α-Amylase Inhibitory Assays

The inhibitory activity of α-amylase ranged from being undetected to an 89.1% inhibition relative to acarbose, and between 0.17 and 101.61% inhibition among assays that did not use acarbose. IC_50_ ranged between 0.038 μg protein/mL and >10.00 mg protein/mL. The details of the α-amylase inhibitory assays used in the research on leguminous antidiabetic peptides are provided in Table 4.

Different enzyme types, enzyme concentrations, sample concentrations, and acarbose concentrations were used. Enzymes were mostly α-amylase from porcine pancreas type VI-B. Some studies used α-amylase from hog pancreas, human saliva, *Bacillus licheniformis*, and *Bacillus subtilis*. Different enzyme types or sources may provide different inhibitory assay results [95], as the inhibitory activity of chickpea legumes in an α-amylase inhibitor was compared against α-amylase enzymes from human saliva (7.0 × 10^−3^ units), *Bacillus subtilis* (120.9 × 10^−3^ units), porcine pancreas (89.1 × 10^−3^ units), maize (5.0 × 10^−3^ units), and *Aspergillus oryzae* (148.4 × 10^−3^ units). The highest inhibitory activity resulted from α-amylase from a porcine pancreas, which was as high as 80.3% [95]. Different concentrations were prepared for each enzyme because enzyme concentration determines the inhibitory activity of the inhibitor. To achieve an inhibitory activity that fell within 0–100% inhibition, the enzyme concentration was adjusted to a suitable range. This is the case in many leguminous antidiabetic peptide studies. The enzyme concentration used varied, including 2 U/mL [16], 10 U/mL [7], 10.8 U/mL [25], 28.57 μg/mL [9], and 0.5 mg/mL [42,62]. An enzyme concentration of 13 U/mL was used in many studies [38,54,55,59,61]. The higher the enzyme concentration used, the higher the inhibitory potential of the sample or the tested inhibitor.

Similar to enzyme concentration, the concentration of a sample or tested inhibitor also determines the inhibitory activity. To achieve inhibitory activity within the 0–100% range, sample concentrations were adjusted. Different sample concentrations were used for different hydrolysates; for example 50 mg protein/mL and 100 mg protein/mL were used for an Alcalase–Flavourzyme–pepsin–pancreatin hydrolysate and a pepsin–pancreatin hydrolysate, respectively [54]. Sample or inhibitor concentrations used among the studies were between 0.05 and 100 mg protein/mL, and a concentration of 1 mg protein/mL was used in many studies [16,55,61,62,64].

Acarbose was used as a positive control at varying concentrations: 1.5–3 μg/mL [9], 0.1–10 mg/mL [7,16], and 1 mM [25,38,55,59,61]. The latter concentration was the most used. The higher the acarbose concentration used, the higher the inhibitory potential of the sample. All studies used starch solution as a substrate at a concentration of 1%.

A synergistic or additive inhibitory effect among peptides with varying sizes was reported [16]. However, it is evident that, across all studies, shorter peptides are more potent inhibitors. Smaller and narrower peptides have more exposure of terminal groups [42] and can easily bind to the catalytic site [9] to inhibit α-amylase activity. Inhibitory peptides from the food matrix have been reported to have two to three amino acids [63,96,97]. These shorter peptides can be produced from protein hydrolysis with diverse enzyme systems [54] and ultrafiltration [65]. Additionally, acidic hydrolysis conditions are likely to produce peptide inhibitors [42].

A lower inhibitory activity is observed in precooked common beans when they are compared to raw samples [59]. Precooking may have denatured naturally occurring α-amylase inhibitors, which are a part of a common bean-defence response mechanism against insects [28,59]. This is in accordance with the findings on naturally occurring <3 kDa peptides in common beans, which have the highest α-amylase inhibitory effect [7].

## 8. α-Glucosidase Inhibitory Assays

α-Glucosidase inhibitory activity ranged between 3.6 and 97.34% inhibition relative to acarbose and between 19.23 and 34.73% inhibition among assays that did not use acarbose. IC_50_ ranged between 0.27 mg protein/mL and >10.00 mg protein/mL. The details of α-glucosidase inhibitory assays used in the research on leguminous antidiabetic peptides are provided in Table 5.

Different enzyme types, enzyme concentrations, sample concentrations, acarbose concentrations, and substrate concentrations were used. In almost all studies, α-glucosidase from *Saccharomyces cerevisiae* was used [12,25,53,54,59,61,64], while the rest originated from rat intestines [9,16]. One study used maltase and sucrase [16]. An enzyme concentration of 1 U/mL was used by most studies. Other studies used 0.1, 0.15, 0.2, and 2 U/mL and 8.33 mg/mL. Sample or inhibitor concentrations were between 1 and 20 mg dry matter/mL and 10 and 200 mg protein/mL. A variation of concentrations used in the studies were adjusted to achieve a comparable inhibitory activity within the 100% range. The higher the enzyme concentration and the lower the sample concentration used, the higher the inhibitory potential of the sample.

Acarbose as a positive control was used at varying concentrations. Many studies set acarbose concentration at 1 mM [25,53,59,61], while others set it at 10 mg/mL [12] and between 0.00625 and 0.125 mg/mL [9]. p-nitrophenyl-α-D-glucopyranoside (PNPG) was used across all studies as substrate, except for one study [16], which used maltose and sucrose. PNPG concentration varied; it was mostly 5 mM [9,25,53,59,64], though other studies used 1 mM [54], 50 mM [65], 100 mM [7], and 1 mg/mL [12]. The higher the acarbose concentration and substrate concentration used, the higher the inhibitory potential of the sample.

The highest α-glucosidase inhibitory activities were observed in samples of peptide fractions with a low molecular weight. Large-molecular-weight peptides are sterically hindered from binding to the enzyme, and thus have a weaker inhibitory activity [65]. However, in the work by Castañeda-Perez et al., the highest inhibitory activity (97.34%) was observed in >10 kDa samples [54]. They argued that the hydrophobic amino acids, which were predominantly present in the fraction, are likely to participate in the inhibition.

Hard-to-cook bean peptide fractions demonstrated a lower inhibitory activity when compared to easy-to-cook beans [7]. Interactions between proteins and other molecules in the seed during postharvest hardening [102] may have effect on the steric hindrance of peptides to interact with the active sites of α-glucosidase during inhibition [7].

## 9. Dipeptidyl Peptidase-IV (DPP-IV) Inhibitory Assays

DPP-IV inhibitory activity ranged between 5 and 96.7% inhibition. IC_50_ ranged between 0.03 and 3 mg protein/mL. DPP-IV inhibitor screening kits were used by all the studies [13,16,25,53,54,55,57,59,61] for leguminous antidiabetic peptides. Details of the DPP-IV inhibitory assays used in the studies are provided in Table 6.

Different enzyme types, enzyme concentrations, sample concentrations, positive control types, and substrate concentrations were used. Purified or recombinant human DPP-IV enzymes or porcine kidney enzymes were used. Enzyme concentrations were 10 ng/mL [53,61], 100 ng/mL [25,55,57,59], 0.26 mU/test well [13,16], and 0.02 U/mL [12]. Sample or inhibitor concentrations were between 0.08 and 5 mg/mL. Diprotin A and sitagliptin were used as positive controls. H-Gly-Pro-p-nitroaniline, Gly-Pro-4-nitroanilide, and Gly-Pro-p-nitroanilide were used at concentrations of 100 μM, 500 μM, and 12 mM, respectively. Most studies reported to have followed the manufacturer’s instructions for the use of the DPP-IV inhibitor kit during the assay.

In contrast to the highest inhibitory activities of α-amylase and α-glucosidase, which were observed in peptide fractions with a lower molecular weight, the highest DPP-IV inhibitory activities were observed in unfractionated hydrolysates. Longer peptides could bind to the secondary binding site of the DPP-IV enzyme [54]. An exception was found only in the study on hard-to-cook common beans of <1 kDa peptide fractions, which showed the highest activity [25].

Lower inhibitory activities were observed in samples hydrolysed with Alcalase at longer hydrolysis times, or with Alcalase and thermolysin hydrolysis when followed by simulated gastro-intestinal digestion (using pepsin and pancreatin) [13,55,57]. Alcalase and the simulated gastro-intestinal digestion that followed Alcalase and thermolysin hydrolysis may have extensively degraded the bean protein to the extent that peptide fractions became no longer potent. Another study [55] also reported that germination did not affect the DPP-IV inhibitory capacity of the common bean. It was concluded that non-germinated and non-hydrolyzed samples had a high DPP-IV inhibitory activity [55].

It is important to note that DPP-IV inhibitory peptide activity depends on the ability to pass through the intestinal epithelium intact and to function at the target site after absorption into the enterocyte [13,59,107]. The absorbability and bioavailability of larger peptides are still being studied. However, there are reports to suggest that larger leguminous, bioactive peptides can be absorbed by the human digestive system [59,108]. The transcellular movement of cell-penetrating peptides (CPPs), the paracellular pathway, and specific transporters (PEPT1 and PEPT2) may be involved in the absorption [59].

## 10. Molecular Mass Profiling

Molecular-mass profiling in the literature for leguminous antidiabetic peptides involves the use of SDS-PAGE, MALDI-TOF/TOF MS, or EASY-MS/MS. The distribution of the protein molecular mass can be described by SDS-PAGE. The molecular mass of each peptide within the protein in the samples and the intensities can be measured using MALDI-TOF/TOF MS or EASY-MS/MS. The results from the latter will further assist in peptide sequencing. MALDI-TOF/TOF MS results are reported only for <1 kDa peptides. One study used EASY-MS/MS [12].

Gel concentrations used in SDS-PAGE were between 4 and 20%, except for one study, which used 5% concentrated and 12% separated polyacrylamide gel [63], and other studies that used 16.5% Tris-Tricine polypeptide-ready gels [55,57]. The molecular markers used were Pre-stained Precision Plus Protein Standard [55,57], Precision Protein Plus Dual Color Standard [25], 10–250 kDa [59], 14.4–116.0 kDa [63], and Carl Roth GmbH and SERVA electrophoresis GmbH [13].

The identification of each noticeable band on SDS-PAGE requires data from other measurements, the protein database, and the literature references. Twelve Mexican and Brazilian common-bean protein identifications [38], observed as SDS-PAGE noticeable bands, were based on SDS-PAGE, tryptic gel LC/MS, Database Uniprot KB20130614, NCBInr 20130614, and the literature [109,110,111,112,113].

The SDS-PAGE of all raw common beans showed noticeable bands at 40–50 kDa, which corresponded to phaseolin. Phaseolin is the main storage protein in *Phaseolus vulgaris* (40–55 kDa) [55,114]. The main storage proteins in *Vigna unguiculata* and *Vigna subterranea* are vicilin (40–70 kDa) [57,115] and glycinine (30–40 kDa). 

The phaseolin in raw common beans remained almost unhydrolyzed by pepsin and pancreatin, while other proteins were digested [38,53,55,59]. Phaseolin protein bands remained noticeable for up to 3 h after germination [55] and fermentation [63].

Phaseolin was hydrolyzed by Alcalase and bromelain, followed by pepsin and pancreatin [25,55]. The early stage of germination (24 h) caused phaseolin to degrade [55] as storage protein was hydrolysed by the endogenous endopeptidase to provide amino acids for developing new tissues [55,116,117]. A longer period of fermentation (>3 h) caused phaseolin protein bands to disappear [63]. Precooking also caused phaseolin protein bands to disappear [59], as protein denatured during thermal and pressure cooking [59,118,119,120].

The SDS-PAGE profile of raw *Vigna unguiculata* and *Vigna subterranea* showed noticeable bands at 50 and 46 kDa, respectively, which corresponded to vicilin. The vicilin fraction remained intact in cowpea beans until 60 h of germination; however, hydrolysis with Alcalase, pepsin, and pancreatin rapidly degraded the fraction [57].

## 11. Peptide Sequence Identification and Bioactivity Prediction

In leguminous antidiabetic peptide studies, peptide sequences in samples were identified by means of HPLC in tandem with MS, such as RP-HPLC-MS/MS, LC-MS-MS/MS, IT-MS-ESI, HPLC-ESI-MS, MS/MS, LCMS, and LC-MS/MS. Data were then processed using Data Analysis^TM^, Mascot Distiller, Xcalibur software v 2.0, MassLynx 4.1 V, and FlexAnalysis 3.4. Further, the data were analysed using data analyser software such as BioTools v 3.1 and v 3.2, Mascot Search, and PEAKS studio v 6.0.

Peptide sequence confirmation was performed using an online protein database. Many studies used the BLAST^®^ tool (http://blast.ncbi.nlm.nih.gov/Blast.cgi (accessed on 4 August 2020)), except for one study, which used the UniProt database (http://www.uniprot.org/ (accessed on 7 February 2019) [13]. Peptide structure and physicochemical properties predictions were aided by an online generator called PepDraw tool (http://www.tulane.edu/~biochem/WW/PepDraw/ (accessed on 4 August 2020).

The potential biological activity of peptides was predicted using BIOPEP database or BioPep tool (http://www.uwm.edu.pl/biochemia/index.php/pl/biopep (accessed on 20 January 2015), Peptide Ranker (http://distilldeep.ucd.ie/PeptideRanker/ (accessed on 20 January 2015), and PeptideDB (http://www.peptides.be/ (accessed on 20 August 2015). Available online databases for potential bioactivities are provided in the form of a list [121]. A list of online tools commonly used in leguminous antidiabetic peptide studies and their functions is provided in Table 7.

Only the selected sample fractions were subjected to peptide sequencing, i.e., samples with the highest inhibitory potential. Moreover, not all peptide fractions detected in the sample were reported as contributing to inhibition. However, in general, many reports indicated that the most important peptides were comprised of amino acids between 3 and 20 residues. The peptides needed to meet several criteria, including a high LC-ESI-MSMS elution profile intensity of ≥50% or even ≥70% [61]; a BioTools Flex score of ≥75 [53]; a PeptideRanker score of >0.80 [42]; a PEAKS Studio Average Local Confidence (ALC) of >60%, and a Pepsite2 *p*-value of <0.05 [62]. LC-ESI-MSMS elution profile intensity ranged from 0 to 100%: the higher the intensity, the more abundant were the peptides present in the sample relative to all the detected peptides. BioTools is a data analysis software used in mass spectrometry to confirm the presence of the detected peptide sequences in a protein of a reference organism. A BioTools Flex score above 70 indicated that the peptide sequence had a higher probability of being a fraction of a protein from the reference organism. PeptideRanker is an online database used to predict peptide bioactivity. A PeptideRanker score of >0.80 meant that the peptide sequence had a higher probability of having the predicted potential bioactivity and having a lower rate of false-positive predictions. PEAKS Studio is a data analysing software used in mass spectrometry to detect the presence of de novo peptide sequences. The confidence levels of peptide novelty based on ALC are classified as very high (>90%), high (80–90%), medium (60–80%), and low (<60%). Pepsite2 is a web server that is used to predict binding sites and analyse the binding mechanisms of bioactive peptides by providing the *p*-value. The optimal *p*-value cut-off is 0.04, and a value above this indicates that the binding prediction is more likely to be accurate.

Peptide sequence identification is crucial in inhibitory peptide studies. Amino acid residues that have an antidiabetic enzyme inhibitory peptide play an important role in how the peptide interacts with the enzymes, i.e., α-amylase, α-glucosidase, and DPP-IV. The types of amino acids and their sequences within a peptide determines the type of interactions or bonds formed between the peptide and the enzymes, as well as the location of the interaction. An interaction at the enzyme catalytic site will potentially result in competitive inhibition or non-competitive and uncompetitive inhibition. Detailed types and sequences of amino acid residues within peptides which affect antidiabetic enzyme inhibitory activity are explained in the next section.

## 12. Molecular Docking and Enzyme Kinetics Study

Software and online tools commonly used in leguminous antidiabetic peptides computational modelling research for molecular docking are ChemBio3D Ultra, Instant MarvinSketch, Maestro, VEGA suites, AutoDock Vina, AutoDock Tools, AutoGrid, Docking Server, PyMol, PLANTS, Discovery Studio (Accelrys Software), Discovery Studio Client (Dasssault Systèmes Biovia Corp ^®^, San Diego, CA, USA), GRAMM-X protein-protein docking web server (http://vakser.compbio.ku.edu/resources/gramm/grammx/ (accessed on 20 August 2020)), and the Rosetta FlexPepDock web server (http://flexpepdock.furmanlab.cs.huji.ac.il/ (accessed on 20 August 2020). Computer-aided techniques to complement empirical methods are powerful tools that allow for the tentative identification of potential leguminous antidiabetic peptides [16,123,125]. A list of software tools used in leguminous antidiabetic peptide studies and their functions is provided in Table 8.

Only selected peptides, which demonstrate good inhibitory potential through empirical evidence, are subjected to computational modelling and enzyme kinetic studies. The selections are based on the results from in vitro, cell work, in vivo and ex vivo experiments, and/or the in silico inhibition activity of the peptides. Additionally, some studies based their selection on the overall occurrence of the peptides in the hydrolysates and from which protein they originated. Peptides from phaseolin and arcelin in common beans and vicilin and lectin in cowpea beans, which were considered main storage proteins, are considered eligible for computational modelling. A list of antidiabetic enzyme inhibitory activities of leguminous protein hydrolysate fractions and peptide sequences is provided in Table 9.

In computer modelling, the inhibitor peptides are analysed using several parameters which include the inhibition constant, energy score (free energy and interface energy), interaction score, distance of interaction, total amino acid interaction, and number of predicted hydrogen bonds. Control molecules are used, i.e., acarbose for α-amylase, α-glucosidase, and sitagliptin for DPP-IV docking analysis. In general, a lower-than-control value indicates better binding conformation and thus better inhibition, except for total amino acid interaction values and the and number of predicted hydrogen bonds, for which higher values indicate a better interaction.

In an enzyme kinetics study, inhibitor peptides were analysed at a minimum of three levels of inhibitor concentrations using Lineweaver–Burk plots (*x*-axis = 1/[S]; *y*-axis = 1/V), in which the Michaelis-Menten constant (*K*_m_) and the maximum velocity of enzymatic activity (*V*_max_) were calculated. The slopes, 1/*K*_m_ values, and 1/*V*_max_ values of the lines from varying inhibitor concentrations defined the type of inhibition. Competitive inhibition was indicated by increasing *K*_m_ values and unchanged *V*_max_ values with the increase in inhibitor concentrations. This type of inhibition is typical with intersecting lines at the y-axis at the same point, indicating unaffected *V*_max_ values even when more inhibitors are present. Non-competitive inhibition is indicated by unchanged *K*_m_ values and reduced *V*_max_ values with increasing inhibitor concentrations. This type of inhibition is typical with intersecting lines at the x-axis at the same point, indicating1/*K*_m_ values unaffected by various inhibitor concentrations. Uncompetitive inhibition is defined by reduced *K*_m_ values and reduced *V*_max_ values with an increase in inhibitor concentrations. For this type of inhibition, lines share the same slope.

Through molecular docking and an enzyme kinetics study, researchers were able to identify the types and locations of chemical interactions between inhibitor peptides and enzymes, as well as the type of enzyme inhibition. The types of interactions between inhibitory peptides and enzymes were hydrogen bonds, hydrophobic interactions, polar interactions, electrostatic interactions, cation π bonds, or van der Waals interactions. The location of the interactions can be inside or outside the enzyme catalytic region, and with the amino acid residues at the side chain or the backbone of the enzyme.

Three structural domains comprise α-amylase (Domain A, residues 1–99, 169–404; Domain B, residues 100–168; Domain C, residues 405–496). Domain A consists of an eight-stranded, parallel β-barrel surrounded by a cylinder of α-helical segments. In this domain, the active sites are located at, ASN197, GLU233, and ASP300 [127]. An α-glucosidase active site identified from the PDB Site Records in the cavity surrounded by the amino acid residues of ALA26, ASP30, SER31, ASN32, ASP33, ASP34, GLY35, TRP36, GLY37, ASP38, LYS40, GLY41, THR83, SER461, PRO448, ALA465, LYS466, PRO467, and TRP468. Enzyme DPP-IV has three binding pockets/sites in both chain A and B. The S1 pocket consists of the residues SER630, ASP708 (ASN710), and HIS740; the S2 pocket involves ARG125, GLU205, and GLU206; and the S3 pocket has SER209, ARG358, and PHE357 [128,129].

Molecular docking of *Phaseolus vulgaris* L. inhibitory peptides on α-amylase were investigated [25,61]. Inhibitory peptides with a good inhibition potential from the first study [61] were AKSPLF, QTPF, and LSKSVL, which interacted with at least two of the three amino acid residues at the catalytic site of the enzyme. The peptides’ interactions with α-amylase occurred through hydrophobic interactions, polar interactions, and hydrogen bonds. Peptides FFL and NEGEAH were reported [25] to have better binding conformations and the lowest-energy interactions with α-amylase when compared to the other peptides detected in their samples. Similar to the first study, all the peptides in this study interacted with at least two amino acid residues of the active site triad of the enzyme. The types of interactions between the peptides and α-amylase reported in the second study were van der Waals interactions, electrostatic interactions, hydrogen bonds, charged interactions, and π interactions. As these peptides interact with the catalytic site of α-amylase, the type of the inhibition is competitive. An enzyme kinetics study of α-amylase by *Pisum sativum* L. inhibitory protein hydrolysates and peptides also reported a similar type of inhibition [9].

It was argued in at least two reports that hydrophobic (A, L, F, V, P, G, and M) and hydrophilic (C, H, and S) amino acids are responsible for human-salivary α-amylase inhibition through hydrophobic interactions and hydrogen bonds at the active site [16,62]. Inhibitory peptides reported in the aforementioned studies [25,61] were mostly composed of hydrophobic and hydrophilic amino acids. Moreover, a review argued that the amino acid residues of H, W, Y, and R are important in α-amylase inhibition [130]. In the same review, authors also mentioned that the inhibition mechanism is initiated when the inhibitory peptides approach the enzyme at the catalytic site, where they cause the formation of an extensive network of interactions and bonds. Enzyme conformational change resulted from the interaction, which disabled the capability of the enzyme to bind with its substrate.

Molecular docking studies between α-glucosidase and soybean [65], common bean [53], or black bean [61] inhibitory peptides have been reported. In the first study, soybean peptides (GSR, EAK) were bound to α-glucosidase outside its active site. The study on soybean peptides [65] found that the interactions were facilitated through van der Waals, anion-π, and hydrogen bonds. The study on common bean peptides [53] reported that polar interactions, hydrophobic interactions, and hydrogen bonds are found between the common bean inhibitory peptides (KTYGL, KKSSG, CPGNK, and GGGLHK) and α-glucosidase. The study on black beans [61] found that the major interactions between black bean peptides (AKSPLF, QTPF, FEELN, and LSKSVL) and α-glucosidase were hydrogen bonds and polar interactions. In the latter studies, peptides bound at the active site of the enzyme, predominantly with ASP34, THR83, and ASN32 at the catalytic site of α-glucosidase. An enzyme kinetic study by Awosika and Aluko [9], however, found non-competitive type of inhibition by *Pisum sativum* L. peptides, similar to the results of Jiang’s group [65].

Amino acid residues with hydroxyl groups (S, T, and Y) or basic groups (K and R) on the side chain at N-terminal, P close at C-terminal, and A and M at the C-terminal position play important roles in α-glucosidase inhibition [131]. The inhibitory peptide sequences reported in the aforementioned studies [53,61,65] are yet to reflect these amino acid positions. However, the major interactions between α-glucosidase and the inhibitory peptides are hydrogen bonds and polar interactions [131]. This is reported in the study on black beans [61]. Hydrophobicity and isoelectric point are unlikely to contribute to α-glucosidase inhibition, while net charges between 0 and +1 are found in the most potent α-glucosidase inhibitor peptides [131].

At least six papers have reported molecular docking studies between DPP-IV and leguminous inhibitory peptides [25,53,55,57,61,126] from soybeans, lupins, common beans, black beans, hard-to-cook beans, and cowpeas. Electrostatic interaction, van der Waals interaction, polar interaction, and hydrogen bonds are the typical interactions reported between DPP-IV and the inhibitory peptides. Only two studies [57,61] reported binding at the DPP-IV active site. In the first study [57], peptide TTAGLLE from a cowpea bean (*Vigna unguiculata*) interacted with the S2 (GLU205 and GLU206) and S3 (SER209, ARG358, and PHE357) active site pockets of DPP-IV. The second study [61] found that inhibitory peptides from black beans (EGLELLLLLLAG, AKSPLF, and FEELN) mainly bind with ASP192, GLU191, and ARG253 at the DPP-IV active site. These amino acid residues of DPP-IV active sites reported by the latter study, however, are different from those reported by Juillerat-Jeanneret [128] and Patel and Ghate [129].

The molecular docking of common-bean inhibitory peptides (KKSSG, CPGNK, GGGLHK, and KTYGL) [53] showed binding with DPP-IV next to the active site (TRP124, GLU237, TYR238, PHE240, PRO249, THR251, VAL252, ARG253, and ALA707). The enzyme kinetic study concluded a competitive type of inhibition. Authors [53] argued that peptides which interacted with the enzyme at several binding sites, and which were able to position in the large cavity of the DPP-IV active structure between the dimer, can cause a blocked access for the substrate to bind with the enzyme, and therefore behave similarly with the inhibition mechanism at active site.

One review concluded that inhibitory peptides IPI, VPL, and LPL are very potent DPP-IV inhibitors [132]. In other reports, dipeptides with P, A, G, S, L, or V at N-terminal are found in DPP-IV inhibitors. Longer peptides—A in the second last position of the N-terminal; P at the first-second-third-fourth N-terminal and flanked by L, V, F, A, and G; hydrophobic amino acids (A, G, I, L, F, P, M, W, and V) at the N-terminal, or approaching the N-terminal or next to the C-terminal; peptides containing W, Y, and F—and the presence of aromatic residue display inhibitory activity against DPP-IV [13,16,54,59,123,126,132,133,134]. The peptides that demonstrated good inhibition potential reported in the six research papers had the hydrophobic amino acid present at a strategic position in their sequence to achieve a good binding conformation with DPP-IV.

## 13. Conclusions

The common bean (*Phaseolus vulgaris*), cowpea bean (*Vigna unguiculata*), Bambara bean (*Vigna subterranea*), soybean (*Glycine max*), and yellow field pea bean (*Pisum sativum*) have been the protein sources in antidiabetic α-Amylase, α-Glucosidase, and DPP-IV inhibitor studies to date. The studies describe sample preparation, protein extraction, hydrolysis, fractionation, inhibitory assays, molecular-mass profiling, peptide sequence identification, the potential bioactivity prediction of the peptide, computational modelling, and enzyme kinetics. Variations in legume cultivars and methods affected the release of peptides. Different methods were used in sample preparation, which include fermentation (t, T), germination (t), and pre-cooking; in protein extraction, including alkaline extraction isoelectric precipitation, phosphate buffer extraction, and water extraction; in protein hydrolysis, enzyme types and combination, enzyme substrate ratio, pH, and time; and in enzyme inhibitory assays’ positive control type and concentration, inhibitor or peptide concentration, and units of inhibitory activity. The categorization of the relative scale of inhibitory activities among legume samples becomes difficult due to these method differences. Peptide fractionations used ultrafiltration membranes from 1 to 100 kDa, ion-exchange chromatography, gel filtration, or reversed-phase high performance liquid chromatography (RP-HPLC). Semi-preparative peptides were obtained using solid-phase methods and standard 9-fluorenylmethyloxycarbonyl (Fmoc) chemistry. Peptide sequences in samples were identified by means of HPLC/MS. Software and online tools were used in bioactivity prediction and computational modelling. The identification of the types and locations of chemical interactions between inhibitor peptides and enzymes and the type of enzyme inhibition were achieved through computational modelling and enzyme kinetic studies.

The highest activity of α-amylase inhibition ranged between 14.9 and 89.1% relative to acarbose and between 57.48 and 101.61% in non-acarbose assays. The highest activity of α-glucosidase inhibition ranged between 22 and 97.34% inhibition relative to acarbose and 34.73% in non-acarbose assays. The highest activity of DPP-IV inhibition ranged between 47.94 and 96.7% inhibition. Peptide inhibitory activity against α-amylase, α-glucosidase, and DPP-IV was demonstrated to depend on its ability to dock at the enzyme catalytic sites. Studies, however, did not indicate the mechanism of how antidiabetic peptides are released into the human digestive system, absorbed into the blood stream, and transported to the target sites.

Further research on leguminous antidiabetic peptides is needed to investigate protein sources from other legumes; the allergenicity and safety of the peptides; the actual sequence of events and the molecular determinants dictating the inhibition mechanism; and the stability, efficacy, and the bioavailability of the peptides. In addition, encapsulation and sensory studies are needed to reduce the typically bitter taste of hydrophobic amino acids; feeding studies using human volunteers are also needed. Lastly, when consumed as legumes in which peptides are still in their parent protein sequence, the yield following peptide release in the human digestive system is still unknown, especially when present in different matrices, macronutrient interactions, and dietary factors. This information is crucial when suggesting the incorporation of legumes into other starch-containing foods or as ingredients in normal diets.

## 14. Future Recommendations

The positive results of the in vitro studies prompt the need for further research on leguminous antidiabetic peptides, as follows:

1. As legumes are an economical dietary protein source, research to explore antidiabetic peptides from other legume sources is needed;
2. Some proteins may cause allergic reactions, and studies on allergenicity and safety of antidiabetic peptides are suggested;
3. The mechanism of how antidiabetic peptides are released into the human digestive system, absorbed into the blood stream, and migrated to the target sites is not fully understood. Moreover, at the molecular level, the actual sequence of events and the molecular determinants dictating the inhibition mechanism are far from being understood, requiring an interdisciplinary approach, such as from nutrition and biomolecular science;
4. When consumed as peptides, the harsh environment in the human digestion tract may cause changes in the peptides that will affect their bioactivity and bioavailability. Hence, peptide stability, efficacy, and bioavailability studies would assist in determining the dose of peptide intake;
5. Low-molecular-weight peptides composed of hydrophobic amino acids are typically bitter, and the application of these peptides in foods without affecting the sensory profile will require a special process such as micro- or nanoparticle encapsulation. Research in this area may offer solutions not only for elucidating sensory aspects, but also bioavailability, bioactivity, and safety;
6. Feeding studies using human volunteers will be required prior to the application of the inhibitory peptides as ingredients in the diets, in functional foods, or as nutraceuticals or pharmaceuticals supplements.

## Figures and Tables

**Table 2:** Protein extraction methods.

Legumes	Authors	Type of Processing	Protein Extraction Method	Sample Preparation	Sample: Water Ratio	Extraction		Precipitation		Method Reference
						pH, T, t	Separation Technique	pH	Separation Technique	
Common bean (*Phaseolus vulgaris* L.)	[59]	Raw and precooked	AE-IP	- Raw sample Or - Blanching - High pressure and thermal cooking - Oven drying	1:10 (beans ground in a commercial blender)	pH 8 (0.1 M NaOH) 35 °C 1 h stirring	- C 5000× *g*, 4 °C, 15 min - S collected - P re-extracted	pH 4.3 (HCl)	- C 5000× *g*, 4 °C, 15 min - Pellet FD, stored at −20 °C	-
Common bean (*Phaseolus vulgaris* L.)	[38]	Raw	AE-IP	- Soaking 16 h - Dehulling (manual) - Grinding (1:10, bean/water)	1:10 (in blender)	pH 8 (0.2 M NaOH) 35 °C 1 h agitation	- C 5000× *g*, 25 °C, 15 min - S collected - P re-extracted	pH 4.3 (HCl)	- C 10,000× *g*, 20 min - P collected, pH adjusted to 8.0 (0.2 M NaOH) to resolubilize protein then pH adjusted to 4.3 to re-P - Pellet VD, stored at −20 °C	[68]
Common bean (*Phaseolus vulgaris* L.)	[53]	Raw	AE-IP	- Soaking 16 h - Dehulling (manual) - Grinding (1:10, bean/water)	1:10 (in blender)	pH 8 (0.2 M NaOH) 35 °C 1 h agitation	- C 5000× *g*, 25 °C, 15 min - S collected - P re-extracted	pH 4.3 (HCl)	- C 10,000× *g*, 20 min - P collected, pH adjusted to 8.0 (0.2 M NaOH) to resolubilize protein then pH adjusted to 4.3 to re-P - Pellet VD, stored at −20 °C	[68]
Black bean (*Phaseolus vulgaris* L.)	[61]	Raw	AE-IP	- Soaking 16 h - Dehulling (manual) - Grinding (1:10, bean/water)	1:10 (in blender)	pH 8 (0.1 M NaOH) 35 °C 1 h stirring	- C 5000× *g*, 25 °C, 15 min - S collected - P re-extracted	pH 4.3 (0.1 M HCl)	- C 10,000× *g*, 4 °C, 20 min - Pellet FD, stored at −20 °C	-
Hard-to-cook bean (*Phaseolus vulgaris* L.)	[25]	Raw	AE-IP	- Soaking 8 h - Dehulling (manual) - Drying 60 °C, 6 h - Milling (20 mesh)	1:10	pH 8.0 (0.2 N NaOH) 35 °C 1 h agitation	- C 5000× *g*, 25 °C, 15 min - S collected - P re-extracted	pH 4.3 (0.1 M HCl)	- C 10,000× *g*, 20 °C, 20 min - P collected, FD, stored at −20 °C	-
Common bean (*Phaseolus vulgaris*)	[55]	Germinated	AE-IP	- Sanitization (sodium hypochlorite 100 mg/kg, 10 min) - Rinsing (3×) - Germination - Dehulling (manual) - Freeze-drying - Milling (20 mesh)	NR	pH 8.0 (0.2 M NaOH) 35 °C 1 h agitation	- C 5000× *g*, 25 °C, 15 min - S collected	pH 4.3 (1.0 M HCl)	- C 10,000× *g*, 20 °C, 20 min - P collected, FD, stored at −20 °C	[68]
Pinto bean (*Phaseolus vulgaris* cv. Pinto)	[42]	Raw	PBE	- Separation from pods - Rinsing - Lyophilisation - Milling (60 mesh)	1:20 (*w*/*v*)	pH 8 ± 0.1 (Phosphate buffer) 25 °C 1 h (250 rpm)	- C 4000× *g*, 30 min - S collected	NA	NA	[69]
Pinto bean (*Phaseolus vulgaris* cv. Pinto)	[62]	Raw	PBE	- Separation from pods - Rinsing - Lyophilisation - Milling (60 mesh)	1:20 (*w*/*v*)	pH 8 ± 0.1 (Phosphate buffer) 25 °C 1 h (250 rpm)	- C 4000× *g*, 30 min - S collected	NA	NA	[69]
Bean (*Phaseolus vulgaris* L. var. Eureka)	[63]	Fermented with *L. plantarum 299v*	WE	- Un-scaling - Soaking 12 h - Milling - Fermentation	NR	NA	- C 8000× *g*, 4 °C, 20 min - S collected, lyophilised, milled	NA	NA	-
Easy-to-cook bean and Hard-to-cook bean (*Phaseolus vulgaris* L.)	[7]	Raw	AE-IP	- Dehulling - Milling (500 μm)	10 g flour in 50 mL water	pH 8.0 (0.1 mol/L NaOH) 35 °C 1 h stirring	- C 5000× *g*, 4 °C, 15 min - S collected - P re-extract	pH 4.3 (diluted HCl)	- C 10,000× *g*, 20 min - P collected, pH adjusted to 8.0 (0.2 M NaOH) to resolubilize protein then pH adjusted to 4.3 to re-P - Pellet VD, stored at −20 °C	[68]
Common bean/Carioca bean (*Phaseolus vulgaris* L. cv Carioca)	[64]	Raw	AE-IP	- Grinding - Sieving (<1 mm)	NR	pH 9.0 (0.1 mol/L NaOH) 1 h stirring	- C 10,000× *g*, 25 °C, 15 min - S collected	pH 4.3 (1 mol/L HCl)	- C (details NR) - P collected, lyophilised	[68,70]
Cowpea bean (*Vigna unguiculata*)	[57]	Germinated	AE-IP	- Sanitization (sodium hypochlorite 100 mg/kg, 10 min) - Rinsing (3×) - Germination - Dehulling (manual) - Freeze-drying - Milling (20 mesh)	NR	pH 8.0 (0.2 M NaOH) 35 °C 1 h agitation	- C 5000× *g*, 25 °C, 15 min - S collected	pH 4.3 (1.0 M HCl)	- C 10,000× *g*, 20 °C, 20 min - P collected, FD, stored at −20 °C	[68]
Cowpea bean (*Vigna unguiculata* L.)	[54]	Raw	AE-IP	Milling	1:6 (*w*/*v*)	pH 11 (T and t NR)	- Sequential sieving (80 and 150 mesh) - Siphon - Soluble protein collected	pH 4.5	- C (details NR) - P collected, lyophilised, stored at 4 °C	[71]
Bambara bean (*Vigna subterranean*)	[13]	Raw	AE-IP	- Grinding (200 μm) - Defatting (hexane/isopropanol, 3/2 ratio) - Air drying - Mixing with 0.17 M NaCl (1:10, *w*/*v*) 35 °C, 150 min	10 g flour in 100 mL 0.17 M NaCl	pH 8.9 4 °C 30 min stirring	- C 2000× *g*, 4 °C, 30 min - S collected - P re-extracted using 100 mL NaCl pH 8.9 - Final S combined with ethanol 95% (2:1, S:ethanol, *v*/*v*)	pH 4.5 washed 2× 15 min	- C 8000× *g*, 4 °C, 20 min - P collected, resuspended in double distilled water maintaining 10% (m/v) total solid - pH adjusted to 7.0 and freeze dried	[72]
Soybean (*Glycine max*)	[16]	Germinated	AE-IP	- Germination - Drying 40 °C, 24 h	NR	pH 9.0 (T and t NR)	NR	pH 4.5	Isoflavones, phenolic, and saponin compounds extracted using 70% ethanol, 1 h agitation, T_room_, until phenolic compounds not detected in alcoholic extract	[73]

AEIP—Alkaline extraction and isoelectric precipitation; WE— water extraction; PBE —phosphate buffer extraction; C— centrifugation; S—supernatant; P—precipitate; VD—vacuum dried; FD—freeze dried; NA—not applicable; NR—not reported.

**Table 3:** Protein hydrolysis methods.

Legumes	Authors	Protein Isolate Solution	Enzymes			Hydrolysis Condition				Hydrolysate Isolation	Cl/Fr/Se/Pf/Sy	Method Reference
			Name	Specification	Ratio	pH	T (°C)	t (min)	Cessation Step			
Common bean (*Phaseolus vulgaris* L.)	[59]	NR	Pepsin	Porcine	1:20 (E/S, *w*/*v*)	2.0	37	120	Continued to pancreatin	Continued to pancreatin		[77]
		From pepsin	Pancreatin	8 × USP	1:20 (E/S, *w*/*w*)	7.5	37	120	75 °C 20 min	C 20,000× *g* 15 min 4 °C S: FD	Clarification: 500 Da (salt elimination)	
Common bean (*Phaseolus vulgaris* L.)	[38]	NR	Pepsin	Porcine 420 U/mg solid	1:20 (E/S, *w*/*v*)	2.0	37	120	Continued to pancreatin	Continued to pancreatin		[77]
		From pepsin	Pancreatin	8 × USP	1:20 (E/S, *w*/*w*)	7.5	37	120	75 °C 20 min	NR	Clarification: 0.45 µm Separation: In-gel tryptic digestion protein analysis	
Common bean (*Phaseolus vulgaris* L.)	[53]	10% (*w*/*v*)	Pepsin	Porcine	1:20 (E/S, *w*/*w*)	2	37	180	Continued to pancreatin	Continued to pancreatin		[77]
		From pepsin	Pancreatin	8 × USP	1:20 (E/S, *w*/*w*)	7.5	37	180	80 °C 20 min	NR	Clarification: 0.45 µm	
Black bean (*Phaseolus vulgaris* L.)	[61]	1:20 (*w*/*v*) protein in water. Autoclaved at 121 °C, 5 min	Protease K	NR	1:20 1:30 1:50 ESR	7.5	37	2 × 603 × 604 × 60	75 °C 20 min	C 20,000× *g* 15 min 4 °C S: FD	Clarification: 500 Da (salt elimination)	-
			Pepsin	NR		2.0	37					
			Trypsin	NR		7.5	37					
			Papain	NR		6.5	60					
			Flavourzyme	NR		8.0	50					
			Thermolysin	NR		8	50					
			Chymotrypsin	NR		7.5	37					
			Alcalase	NR		7.0	50					
		1:20 (*w*/*v*) Alcalase hydrolysate in water	Pepsin	NR	1:20 (E/S, *w*/*w*)	2.0	37	2 × 60	Continued to pancreatin	Continued to pancreatin		
		From pepsin	Pancreatin	NR	1:20 (E/S, *w*/*w*)	7.5	37	2 × 60	75 °C 20 min	C 20,000× *g* 15 min 4 °C S: FD	Clarification: 500 Da (salt elimination)	
Hard-to-cook bean (*Phaseolus vulgaris* L.)	[25]	1:10 (*w*/*v*) Protein in water. Incubated at pH 7.0, 70 °C, 15 min	Alcalase	*Bacillus licheniformis*	1:17 ESR	8 (0.5 N NaOH)	50	NR	75 °C 20 min Continued to pepsin	C 14,000× *g* 30 min 4 °C S: FD	Clarification: 3 kDa (salt removal)	[68,77]
			Bromelain	Pineapple	1:17 ESR	7 (0.5 N NaOH)	45	NR	75 °C 20 min Continued to Pepsin	C 14,000× *g* 30 min 4 °C S: FD		
		From Alcalase and bromelain	Pepsin	662 units/mg	1:20 ESR	2.0	37	3 × 60	Continued to pancreatin	Continued to pancreatin		
		From pepsin	Pancreatin	8 × USP	1:20 ESR	7.5	37	3 × 60	75 °C 20 min	C 20,000× *g* 15 min 4 °C	Fractionation: 1, 3, 5, and 10 kDa	
Common bean (*Phaseolus vulgaris*)	[55]	8:100 (*w*/*v*) Incubated at pH 8.0 (0.5 M NaOH), 50 °C, 10 min	Alcalase	*Bacillus licheniformis* 2.4 AU/g	0.75 AU/g of protein	8.0 (0.5 M NaOH)	NR	0 1 × 60 2 × 60 3 × 60 4 × 60	0.1 M HCl 1.2 mL Continued to pepsin	C 14,000× *g* 30 min 10 °C S: FD	Clarification: 0.8 kDa (salt elimination)	-
		From Alcalase	Pepsin	Porcine	1:20 (E/S, *w*/*w*)	2.0	37	120	Continued to pancreatin	Continued to pancreatin		[77]
		From pepsin	Pancreatin	NR	1:20 (E/S, *w*/*w*)	7.5	37	120	75 °C 20 min	C 20,000× *g* 15 min 4 °C S: FD		
Pinto bean (*Phaseolus vulgaris* cv. Pinto)	[42]	1:10 (*w*/*v*) Protein in phosphate buffer	Protamex	NR	1:10 1:30 1:50 (E/S, *w*/*v*)	6.5 7.5 8.5	50	30 60 90	95 °C 30 min	C 15 min 4 °C S stored at −20 °C	Fractionation: 3, 10, 30, and 50, 100 kDa	-
Pinto bean (*Phaseolus vulgaris* cv. Pinto)	[62]	1:10 (*w*/*v*) Protein in phosphate buffer	Protamex	NR	1:10 1:30 1:50 (E/S, *w*/*v*)	6.5 7.5 8.5	50	30 60 90	95 °C 30 min	C 15 min 4 °C S stored at −20 °C	Fractionation: 3, 10, 30, 50, and 100 kDa Synthesis: Mimotopes, Clayton, VIC, Australia	-
Bean (*Phaseolus vulgaris* L. var. Eureka)	[63]	4% (*w*/*v*) protein in salt solution (7 mM NaHCO_3_ and 0.35 mM NaCl) Incubated at 37 °C, 5 min	α-Amylase	Hog pancreas 50 U/mg	1:10 ESR	NR	37	10	Continued to pepsin	Continued to pepsin		[78,79]
		From α-amylase	Pepsin	Porcine gastric mucosa 250 units/mg	1:100 ESR	2.5 (1 M HCl)	37	120	Continued to pancreatin	Continued to pancreatin		
		From pepsin	Pancreatin (0.7%) and bile extract (2.5%)	Porcine pancreas	1:2.5 ESR	Neutral (1 M NaOH)	37	60	100 °C 5 min	NR	Fractionation: 3.5 and 7.0 kDa Separation: Sephadex G10	
Easy-to-cook bean and hard-to-cook bean (*Phaseolus vulgaris* L.)	[7]	NR	Pepsin	NR	1:20 (E/S, *w*/*w*)	2.0	37	120	Continued to pancreatin	Continued to pancreatin		[80]
		From pepsin	Pancreatin	NR	1:20 (E/S, *w*/*w*)	7.5	37	120	80 °C 20 min	C 5000× *g* 10 min 4 °C S: FD	Clarification: 0.45 µm Fractionation: 3 and 10 kDa	
Common bean/Carioca bean (*Phaseolus vulgaris* L. cv Carioca)	[64]	100 mg/mL	Simplex centroid mixture design of: Flavourzyme-Alcalase-Neutrase	Flavourzyme^TM^ 500 L *Aspergillus oryzae* Alcalase^TM^ 2.4 L *Bacillus licheniformis* Neutrase^TM^ 0.8 L *Bacillus amyloliquefaciens*	50 U/mL	7	50	120	100 °C 20 min	C 10,000× *g* 20 min 5 °C S: FD		-
Cowpea bean (*Vigna unguiculata*)	[57]	8:100 (*w*/*v*) Incubated at pH 8.0 (0.5 M NaOH), 50 °C, 10 min	Alcalase	*Bacillus licheniformis* 2.4 AU/g	0.75 AU/g of protein	8.0 (maintained by 0.5 M NaOH)	NR	0 1 × 60 2 × 60 3 × 60 4 × 60	0.1 M HCl 1.2 mL Continued to pepsin	C 14,000× *g* 30 min 10 °C S: FD	Clarification: 0.8 kDa (salt elimination)	-
		From Alcalase	Pepsin	Porcine	1:20 (E/S, *w*/*w*)	2.0	37	120	Continued to pancreatin	Continued to pancreatin		[77]
		From pepsin	Pancreatin	NR	1:20 (E/S, *w*/*w*)	7.5	37	120	75 °C 20 min	C 20,000× *g* 15 min 4 °C S: FD		
Cowpea bean (*Vigna unguiculata* L.)	[54]	4% (*w*/*v*)	Alcalase	Alcalasa^®^ *Bacillus licheniformis*	0.3 AU/g protein isolate	8.0	50	45	Continued to pepsin	Continued to pepsin		[81]
		4% (*w*/*v*)	Flavourzima	Flavourzima^®^ *Aspergillus oryzae*	50 UAPL/g protein isolate	7.0	50	45	Continued to pepsin	Continued to pepsin		
		From Alcalase and Flavourzima	Pepsin	Sigma P70007 Porcine gastric mucosa	1:10 ESR	2	37	45	Continued to pancreatin	Continued to pancreatin		
		From pepsin	Pancreatin	Sigma 1750 Pig pancreas	1:10 ESR	7.5	37	45	NR	NR	Fractionation: 1, 3, 5, and 10 kDa	
Bambara bean (*Vigna subterranean*)	[13]	5% (*w*/*v*) protein in double-distilled water	Alcalase	*Bacillus licheniformis* ≥2.4 U/g protein	4% ESR	7 (adjusted every 30 min, 0.5 M NaOH)	NR	24 × 60	95 °C 5 min	C 8000× *g* 10 min 4 °C S: FD		[82]
			Trypsin	Porcine pancreas 1.5 U/g protein	1% ESR	7 (adjusted every 30 min, 0.5 M NaOH)	55	24 × 60	95 °C 5 min	C 8000× *g* 10 min 4 °C S: FD		
			Thermolysin	*Geobacillus stearothermophilus* 0.03–0.17 U/g protein	1% ESR	8 (adjusted every 30 min, 0.5 M NaOH)	70	24 × 60	95 °C 5 min	C 8000× *g* 10 min 4 °C S: FD	Separation: RP-HPLC	
		20 mg hydrolysate/mL in 0.01 M HCl	Pepsin	Porcine gastric mucosa 3200–4500 U/mg protein	0.04 mg/mg ESR	2.10	37	30		Continued to α-chymotrypsin and trypsin		[83]
		From pepsin	α-Chymotrypsin	Bovine pancreas ≥0.04 U/g protein	0.02 mg/mg ESR	7.5				Continued to pancreatin		
			Trypsin	Porcine pancreas 1.5 U/g protein	0.08 mg/mg ESR	7.5						
		From α-chymotrypsin and trypsin	Pancreatin	NR	NR	7.5	37	90	100 °C 5 min	C 8000× *g* 10 min 4 °C S: FD		
Soybean (*Glycine max*)	[16]	5% (*w*/*v*) protein in distilled water	Pepsin	Porcine gastric mucosa 250 units/mg solid	4% (*w*/*v*, protein basis)	2.0 (1N HCl)	37	60	Continued to pancreatin	Continued to pancreatin		[84]
		From pepsin	Pancreatin	Porcine pancreas 8 × USP	4% (*w*/*v*, protein basis)	7.5 (1 N NaOH)	37	120	100 °C 10 min	C 16,000× *g* 10 min S: FD	- Fractionation: 5 and 10 kDa - Separation: RP-HPLC	
Soybean	[65]	6:10 (*w*/*v*) protein in distilled water Ultrasonication (65 kHz, 245 W), T_room_, 20 min Cooled to 37 °C. Diluted to 1.0% pH adjusted to 8.0 (1 M NaOH), incubated at 37 °C for 5 min	Trypsin	6400 U/g	NR	8.0 (1 M NaOH)	37	22	90 °C 10 min	C 8000× *g* 25 min	- Clarification: 0.45 µm - Fractionation: 5 kDa - Separation: ion-exchange chromatography, Sephadex G25, RP-HPLC - Synthesis: Solid-phase method and standard Fmoc chemistry	[85,86]
Soy	[87]	2% (*w*/*v*) protein in distilled water Heated to 100 °C for 10 min. Let cool	Alkaline Protease	200 U/mg	6000 U/g protein content in slurry	9	50	Until DH stable	100 °C 10 min Continued to pepsin	C 4.436× *g* 10 min Stored at −20 °C		-
			Papain	800 U/mg		6.5	60	Until DH stable				-
			Trypsin	NR		7	37	Until DH stable				-
		10 mg/mL hydrolysate in deionized water	Pepsin	Porcine gastric mucosa 500/mg	2.5 U/mg substrate	2 (1 M HCl)	37 (stirring waterbath)	2 × 60	Continued to pancreatin	Continued to pancreatin		[88]
		From Pepsin	Pancreatin	Porcine pancreas	4:100 ESR	7.2 (1 M NaOH)	37	2 × 60	95 °C 20 min	C 8000 rpm 10 min	Isolation: DEAE-52, Sephadex G-15	
Yellow field pea (*Pisum sativum* L.)	[9]	5% protein in double-distilled water	Alcalase	NR	4:100 ESR	NR	NR	4 × 60	pH 5.0 (2 M NaOH, 2 M HCl) 95 °C 15 min	C 10,000× *g* 15 min 4 °C S: FD	Fractionation: 1, 3, 5, and 10 kDa	-
			Chymotripsin	NR		NR	NR					
			Pepsin	NR		NR	NR					
			Trypsin	NR		NR	NR					

ESR—enzyme substrate ratio; Cl—clarification; Fr —fractionation; Se—separation; Pf—purification; Sy—synthesis; C—centrifugation; S—supernatant; FD—freeze dried; NR—not reported.

**Table 4:** α-Amylase Inhibitory Assay.

Legumes	Authors	α-Amylase Type	Volume, Activity, Concentration, pH					Results Range		Method Reference
			Enzyme	Inhibitor	Control (Acarbose)	Substrate (Starch)	Buffer	% Inhibition	IC_50_	
Common bean (*Phaseolus vulgaris* L.)	[59]	Porcine pancreas type VI-B	500 µL 13 U/mL buffer	500 µL	500 µL 1 mM	500 µL 1% soluble starch in buffer	0.02 M Na-phosphate buffer, pH 6.9	Hydrolysates: ~4–36% rel ac/mg protein	-	-
Common bean (*Phaseolus vulgaris* L.)	[38]	Porcine pancreas type VI-B	500 µL 13 U/mL buffer	500 µL	500 µL 1 mM	500 µL 1% soluble starch in buffer	0.02 M Na-phosphate buffer, pH 6.9	Hydrolysates: 2.5–14.9% rel ac/mg BPI	-	[59]
Black bean (*Phaseolus vulgaris* L.)	[61]	Porcine pancreas type VI-B	500 µL 13 U/mL buffer	500 µL 1 mg DM/mL buffer	500 µL 1 mM	500 µL 1% soluble starch in buffer	0.02 M Na-phosphate buffer, pH 6.9	Hydrolysates: 13.0–61.8% inhibition/mg dry hydrolysate	-	[10]
Hard-to-cook bean (*Phaseolus vulgaris* L.)	[25]	*B. subtilis*	500 µL 10.8 U/mL	500 µL 100 µg/mL	500 µL 1 mM	500 µL 1% starch in buffer	0.02 mM phosphate buffer with 6 mM NaCl, pH 6.9	Hydrolysates: ~15–50% rel ac Peptide fractions: ~5–51% rel ac	-	-
Common bean (*Phaseolus vulgaris*)	[55]	Porcine pancreas type VI-B	500 µL 13 U/mL buffer	500 µL 1 mg/mL	500 µL 1 mM	500 µL 1% soluble starch in buffer	0.02 M Na-phosphate buffer, pH 6.9	7.61–30.88% rel ac	-	-
Pinto bean (*Phaseolus vulgaris* cv. Pinto)	[42]	NR	500 µL 0.5 mg/mL buffer	100 µL	Control: enzyme, starch, without inhibitor Blank: starch, inhibitor, without enzyme	500 µL 1% starch in buffer	0.02 M Na-phosphate buffer in 6 mM NaCl, pH 6.9	Hydrolysates: 15.78–57.48% Peptide fractions: 0.17–62.1%	-	[89]
Pinto bean (*Phaseolus vulgaris* cv. Pinto)	[62]	Human saliva	500 µL 0.5 mg/mL buffer	100 µL 1 mg/mL	Control: enzyme, starch, without inhibitor Blank: starch, inhibitor, without enzyme	500 µL 1% (*w*/*v*) starch in buffer	0.02 M, Na-phosphate buffer in 6 mM NaCl, pH 6.9	Peptide fractions: ~5–58% inhibition/100 µg pinto bean peptides	Synthetic peptide fractions: 23.33–10.03 mM	[89]
Bean (*Phaseolus vulgaris* L. var. Eureka)	[63]	Hog pancreas (50 U/mg)	0.25 mL	0.25 mL	Enzyme, starch, without inhibitor	0.5 mL 1% (*w*/*v*) soluble starch in buffer	100 mM phosphate buffer containing 6 mM NaCl, pH 7.0	-	Peptide fractions: 2.12– 0.038 µg/mL	[90]
Easy-to-cook bean and hard-to-cook bean (*Phaseolus vulgaris* L.)	[7]	Porcine pancreas	500 µL 10 U/mL buffer	500 µL 10 mg/mL	500 µL 10 mg/mL	500 µL 1% starch in buffer	50 mmol/L Na-phosphate buffer, pH 6.9	Naturally occurring peptide fractions: 3.0–89.1% Peptide fractions in hydrolysates: 1.3–53.4% Acarbose 32.8%	-	[59,91]
Common bean/Carioca bean (*Phaseolus vulgaris* L. cv Carioca)	[64]	*B. licheniformis* (Termamyl^®^ 2X)	0.5 mL in buffer	0.5 mL 1 mg/mL	Enzyme, starch, distilled water	0.5 mL 1% (*w*/*v*) starch in buffer	50 mmol/L phosphate buffer, pH 7	Non hydrolysed protein: 4.67% Hydrolysates: 30.05–101.61%	-	[91,92]
Cowpea bean (*Vigna unguiculata* L.)	[54]	Porcine pancreas type VI-B	200 µL 13 U/mL buffer	200 µL For PP: 100 mg/mL water For AF-PP: 50 mg/mL water	Details not reported	200 µL 1% starch in buffer	20 mM Na-phosphate buffer, pH 6.9	Hydrolysates and peptide fractions: 24.39–96.81% Acarbose: 98.41%	-	[59]
Soybean (*Glycine max*)	[16]	Porcine pancreas	100 µL 2 U/mL buffer	50 µL Hydrolysate: 0.2–4 mg/mL Peptide fractions: 1 mg/mL	50 µL 0.1–1.3 mg/mL Negative control: 50 µL distilled water	100 µL 1% potato soluble starch in buffer	0.02 M Na-phosphate buffer, pH 6.9	Peptide fractions: ~84% not detected, relative to negative control	Hydrolysate and peptide fractions: >10.00– 1.7 mg peptide/mL Acarbose: 0.16 mg acarbose /mL	[93]
Yellow field pea (*Pisum sativum* L.)	[9]	Porcine pancreas	100 µL 28.57 µg/mL buffer	100 µL 50–225 µg/mL buffer	100 µL 1.5–3 µg/mL buffer	100 µL 1 g/100 mL starch in buffer	0.02 M Na-phosphate buffer, pH 6.9	Hydrolysates: ~7–30% Peptide fractions: ~2–30% Acarbose: ~45–74%	-	[94]

Inhibitors—protein isolate, protein hydrolysate, peptide fractions; rel ac—relative to acarbose; BPI—bean protein isolate; AF—Alcalase–Flavourzyme; PP—pepsin–pancreatin; NR—not reported.

**Table 5:** α-Glucosidase Inhibitory Assay.

Legumes	Authors	α-Glucosidase Type	Volume, Activity, Concentration, pH					Results Range		Method Reference
			Enzyme	Inhibitor	Control (Acarbose)	Substrate	Buffer	% Inhibition	IC_50_	
Common bean (*Phaseolus vulgaris* L.)	[59]	*S. cerevisiae*	100 µL 1 U/mL buffer	50 µL	50 µL 1 mM	50 µL PNPG 5 mM in buffer	0.1 M Na-phosphate buffer, pH 6.9	Hydrolysates: ~48–68% inh AC/mg protein	-	-
Common bean (*Phaseolus vulgaris* L.)	[53]	*S. cerevisiae*	100 µL 1 U/mL buffer	50 µL 1 mg DW/mL	50 µL Acarbose 1 mmol/L	50 µL PNPG 5 mmol/L in buffer	0.1 mol/L Na-phosphate buffer, pH 6.9	Hydrolysates: 46.90–50.10% Peptides: 36.30–49.34%	-	-
Black bean (*Phaseolus vulgaris* L.)	[61]	*S. cerevisiae*	100 µL 1 U/mL buffer	50 µL 1 mg DM/mL buffer	50 µL 1 mmol/L	50 µL PNPG 1 mM in buffer	0.1 M Na-phosphate buffer, pH 6.9	Hydrolysates: 3.6–78.4% inhibition/mg dry hydrolysate	-	[10]
Hard-to-cook bean (*Phaseolus vulgaris* L.)	[25]	*S. cerevisiae*	100 µL 1.0 U/mL buffer	50 µL	50 µL 1 mM	50 µL PNPG 5 mM in buffer	0.1 M phosphate buffer, pH 6.9	Hydrolysates: ~37–65% rel ac Peptide fractions: ~50–74% rel ac	-	[92]
Easy-to-cook bean and hard-to-cook bean (*Phaseolus vulgaris* L.)	[7]	NR	60 µL 2 U/mL	60 µL 10 mg protein/mL	60 µL 10 mg/mL	60 µL PNPG 0.1 mol/L	0.5 mol/L phosphate buffer, pH 6.8	Naturally occurring peptide fractions: 35.9–89.2% Peptide fractions in hydrolysates: 22.9–53.5% Acarbose 76.0%	-	[59]
Common bean/Carioca bean (*Phaseolus vulgaris* L. cv Carioca)	[64]	*S. cerevisiae*	100 µL 0.1 U/mL	50 µL 1 mg/mL buffer	Distilled water	50 µL PNPG 5 mmol/L	50 mmol/L phosphate buffer, pH 7	Non-hydrolysed protein: 19.23% Hydrolysates: 8.67–34.73%	-	[92]
Cowpea bean (*Vigna unguiculata* L.)	[54]	*S. cerevisiae* (SLBP0778V)	20 µL 2 U/mL buffer	20 µL For PP: 200 mg protein/mL water For AF-PP: 10 mg protein/mL water	Details not reported	20 µL PNPG 1 mM	50 mM K-phosphate buffer, pH 6.8	Hydrolysates and peptide fractions: 4.069–97.34% Acarbose: 90.18%	-	[59]
Soybean (*Glycine max*)	[16]	Rat intestine	50 µL 1 U/mL buffer	100 µL 1–10 mg/mL	100 µL Negative control: distilled water	50 µL Maltose 2 mM	0.1 M maleat buffer, pH 6.9	Peptide fractions (substrate maltose): ~20–32%	Hydrolysate and peptide fractions (substrate maltose): >10.00–2.56 mg/mL Acarbose: 0.07 mg/mL	[93]
			50 µL 1 U/mL buffer	100 µL 1–10 mg/mL	100 µL Negative control: distilled water	50 µL Sucrose 20 mM	0.1 M maleat buffer, pH 6.9	Peptide fractions (substrate sucrose): ~8–22%	Hydrolysate and peptide fractions (substrate sucrose): 5.27–1.23 mg/mL Acarbose: 0.03 mg/mL	-
Soybean	[65]	NR	0.2 mL 0.15 U/mL	0.1 mL	Control 1:bBuffer, substrate, enzyme Control 2: buffer, enzyme Control 3: enzyme, inhibitor	0.2 mL PNPG 50 mM in buffer	0.1 M K-phosphate buffer, pH 6.8	-	Hydrolysate: 1.93 mg/mL Peptides < 5 kDa: 0.27 mg/mL Peptides > 5 kDa: 3.31 mg/mL Glu-Ser-Arg: 20.4 μM Glu-ala-Lys: 520.2 μM	[98]
Soy	[12]	*S. cerevisiae*	10 µL 0.2 U/mL buffer	50 µL	10 mg/mL buffer	50 µL PNPG 1 mg/mL buffer	0.2 M Na-phosphate buffer	Hydrolysates: ~30–80% Alkaline protease H1 Fraction from DEAE-52: 87.10 ± 2.70% Alkaline protease H1 fraction from DEAE-52 then H-12 fraction from Sephadex G-15: 95.35 ± 2.70%	Alkaline protease hydrolysate: 4.94 ± 0.07 mg/mL Acarbose: 0.52 ± 0.05 mg/mL	[99]
Yellow field pea (*Pisum sativum* L.)	[9]	Rat intestinal acetone powder	50 µL 8.33 mg/mL buffer	50 µL 5–20 mg/mL buffer	50 µL 0.00625–0.125 mg/mL buffer	100 µL PNPG 5 mM in buffer	0.1 M Na-phosphate buffer, pH 6.9	Hydrolysates: ~8–47% Peptide fractions: ~6–53% Acarbose: ~45–67%	-	[8,100,101]

Inhibitors—protein isolate, protein hydrolysate, peptide fractions; inh AC—relative to acarbose; DW — dry weight; PNPG—p-nitrophenyl-α-D-glucopyranoside; NR—not reported.

**Table 6:** DPP-IV Inhibitory Assay.

Legumes	Authors	DPP-IV Inhibitor Kit	DPP-IV Type	Volume, Activity, Concentration, pH						Results Range		Method Reference
				Enzyme	Inhibitor	Control	Blank	Substrate	Assay Buffer	% Inhibition	IC_50_	
Common bean (*Phaseolus vulgaris* L.)	[59]	DPP-IV Glo Protease assay (Promega, G8351)	Purified DPP-IV, human enzyme	10 µL 100 ng/mL	40 µL 1 mg DW/mL	40 µL Enzyme control	50 µL Assay buffer	50 µL DPP-IV Glo reagent	100 mM Tris pH 8.0 200 mM NaCl 1 mM EDTA	-	Hydrolysates: ~1–0.1 mg protein/mL	-
Common bean (*Phaseolus vulgaris* L.)	[53]	DPP-IV Glo Protease assay (Promega, G8351)	Purified DPP-IV, human enzyme (D4943)	10 µL 10 ng/mL	40 µL 1 mg DW/mL	40 µL Enzyme control	50 µL Assay buffer	50 µL DPP-IV Glo reagent	100 mmol/L Tris pH 8.0 200 mmol/L NaCl 1 mmol/L EDTA	-	Hydrolysates: 0.33–0.14 mg DW/mL Peptides: 0.87–0.03 mg DW/mL Diprotin A: 0.02 mg DW/mL	-
Black bean (*Phaseolus vulgaris* L.)	[61]	DPP-IV Glo Protease assay (Promega, G8351)	Purified DPP-IV, human enzyme	10 µL 10 ng/mL	40 µL 1 mg DW/mL	40 µL Enzyme control	50 µL Assay buffer	50 µL DPP-IV Glo reagent	100 mM Tris pH 8.0 200 mM NaCl 1 mM EDTA	Hydrolysates: 13.9–96.7%	-	-
Hard-to-cook bean (*Phaseolus vulgaris* L.)	[25]	DPP-IV (Sigma Aldrich, protocol SSGPNA01)	DPP-IV, porcine kidney	100 ng/mL	100 µg/mL	NR	NR	500 µM Gly-Pro-4-Nitroanilide	100 mM Tris pH 8.0	Hydrolysates: ~5–55%	-	[103]
Common bean (*Phaseolus vulgaris*)	[55]	DPP-IV Glo Protease assay (Promega, G8351)	Purified DPP-IV, human enzyme (≥1.0 U/vial)	10 µL 100 ng/mL	40 µL 0.1–4.0 mg/mL buffer	Enzyme control: 40 µL	50 µL Assay buffer	50 µL DPP-IV Glo reagent	100 mM Tris pH 8.0 200 mM naCl 1 mM EDTA	-	Hydrolysates: ~3.3–0.75 mg hydrolysate/mL	-
Cowpea bean (*Vigna unguiculata*)	[57]	DPP-IV Glo Protease assay (Promega, G8351)	Purified DPP-IV, human enzyme	10 µL 100 ng/mL	40 µL 0.1–4.0 mg/mL buffer	Enzyme control: 40 µL	50 µL Assay buffer	50 µL DPP-IV Glo reagent	100 mM Tris pH 8.0 200 mM NaCl 1 mM EDTA	-	Hydrolysates: ~3.0–0.5 mg hydrolysate/mL	
Cowpea bean (*Vigna unguiculata* L.)	[54]	DPP-IV (Sigma-Aldrich, MAK203)	NR	NR	NR	Sitagliptin	NR	NR	NR	Hydrolysates and peptide fractions: 67.65–85% Sitagliptin: 97.77%	-	-
Bambara bean (*Vigna subterranean*)	[13]	DPP-IV Drug Discovery Kit (Enzo Life Sciences)	Recombinant-soluble human DPP-IV	15 µL 0.26 mU/test well	50 µL/well 1 mg/mL	Diprotin A	NR	50 µL 100 µM H-Gly-Pro-p-nitroaniline in assay buffer	NR	Hydrolysates: 7.981 ± 0.240–44.253 ± 1.327% at 1 mg/mL Simulated GI digesta: 8.996 ± 0.043–29.276 ± 0.878% at 1 mg/mL	Hydrolysates: 1.733→2.5 mg/mL	[104]
Soybean (*Glycine max*)	[16]	DPP-IV Drug Discovery Kit (Enzo Life Sciences)	Recombinant-soluble human DPP-IV	15 µL 0.26 mU/test well	50 µL/well 0.08–5 mg/mL	Positive control: 50 µL/well Diprotin A 0.78–50 µM	NR	50 µL 100 µM H-Gly-Pro-p-nitroaniline in assay buffer	NR	-	Hydrolysate and peptide fractions: 2.21–0.91 mg/mL Diprotin A: 0.003 mg/mL	[105]
Soy	[12]	-	DPP-IV from human	50 µL 0.02 U/mL buffer	25 µL	Buffer to replace enzyme	Buffer to replace inhibitor and enzyme	25 µL 12 mM Gly-Pro-p-nitroanilide	Tris buffer pH 8.0	Hydrolysates: ~40–47%	Alkaline protease hydrolysate: 2.73 ± 0.08 mg/mL	[106]

Inhibitors—protein isolate, protein hydrolysate, peptide fractions; NR—not reported.

**Table 7:** Online tools used in leguminous antidiabetic peptides studies.

Name of Online Tools	Link	Function	References
RSCB PDB	http://www.rcsb.org/pdb/home/home.do (accessed on 5 August 2020)	To retrieve the 3D crystal structure of enzymes.	[25,53,55,57,61,62,65,122]
UniProt	http://expasy.org/ (accessed on 5 August 2020)	To provide access to protein databases and software tools.	[122,123]
BLAST	https://blast.ncbi.nlm.nih.gov/Blast.cgi (accessed on 5 August 2020)	To confirm regions of similarity between biological sequences.	[25,38,53,55,57,59,61]
PeptideDB	http://www.peptides.be (accessed on 5 August 2020)	To validate the novelty of peptides.	[62]
PepDraw	http://www.tulane.edu/~biochem/WW/PepDraw/ (accessed on 5 August 2020)	To predict peptide structures and physicochemical properties.	[16,38,53,55,57,59,122]
I-TASSER	https://zhanglab.dcmb.med.umich.edu/I-TASSER/ (accessed on 5 August 2020)	To predict protein structure and structure-based functions.	[122,124]
BIOPEP	http://www.uwm.edu.pl/biochemia/index.php/pl/biopep (accessed on 5 August 2020)	To predict potential biological activities of peptides.	[16,25,38,53,55,57,59,61,62,122]
PeptideRanker	http://distilldeep.ucd.ie/PeptideRanker/ (accessed on 5 August 2020)	To predict peptide bioactivity potential.	[42,62]
Pepsite2	http://pepsite2.russelllab.org/ (accessed on 5 August 2020)	To predict the binding sites and analyze the binding mechanisms of bioactive peptides.	[62]
GRAMM-X	http://vakser.compbio.ku.edu/resources/gramm/grammx/ (accessed on 5 August 2020)	To simulate molecular docking.	[25,55]
Rosetta FlexPepDock	http://flexpepdock.furmanlab.cs.huji.ac.il/ (accessed on 5 August 2020)	To refine the peptide–protein docking complex models.	[25,55]

**Table 8:** Software tools used in leguminous antidiabetic peptides studies.

Name of Software	Function	References
ChemBio3D Ultra	To convert 2D peptides to 3D	[65]
Instant MarvinSketch	To design peptides	[53]
Maestro	To model protein structure	[122,124]
VEGA suites	To build a canonical α-helix of peptides	[126]
AutoDock Vina	- To investigate molecular docking - To perform docking calculation	[65,122]
AutoDock Tools	- To generate docking input files - To add essential hydrogen atoms, Kollman united atom type charges, and solvation parameters - To assign flexible torsions, charges, and grid size	[53,61,65,122,124]
AutoGrid	To generate affinity maps and spacing	[53]
DockingServer	To perform docking calculation	[53,61,124]
PyMol	To visually analyze results from AutoDock Vina	[65]
PLANTS	To simulate docking	[126]
Discovery Studio (Accelrys Software)	- To run loop refinement and energy minimization - To visualize the selected binding pose with the lowest binding energy obtained from Vina - To delete water molecules, attached ligands, and monomeric units of DPP-IV; to add hydrogen atoms and a CHARMM36 force field; to draw peptides; to select docking conformation output from GRAMM-X and Rosetta FlexPepDock	[25,55,57,61,122,124]
Discovery Studio Client (Dassault Systèmes Biovia Corp ^®^)	- To visualize the selected binding pose with the lowest binding energy obtained from Vina	[122]

**Table 9:** Antidiabetic enzyme inhibitory activity of leguminous protein hydrolysate fractions and peptide sequences.

Protein Source	Most Important Peptides	Protein Hydrolysate Fraction	Peptide Sequence	Inhibitory Activities				Authors
				α-Amylase	α-Glucosidase	DPP-IV	Others	
Soybean (*Glycine max*)	Potential α-amylase, α-glucosidase, DPP-IV inhibitor peptides: subfractions F1, F2, and F3 collected by RP-HPLC from 5–10 kDa fraction obtained from 6-day germinated soybean protein digest	5–10 kDa, F1	NNDDRDS, VVNPDNNEN, LSSTEAQQS, NAENNQRN, IKSQSES, EEPQQPQQ, GQSSRPQD, LAGNQEQE, NLKSQQA, QEPQESQQ, SQRPQDRHQ, QQQQQGGSQSQ, QQQQQGGSQSQKG, PETMQQQQQQ, SDESTESETEQA	85%	Maltase: 28% Sucrase: 22%	IC_50_: 0.8 mg/mL	NR	[16]
		5–10 kDa, F2	RNLQGENEEEDSGA, VTRGQGKV, KKGVIT, IMSDESTESETEQA	20%	Maltase: 21% Sucrase: 21%	IC_50_: 0.75 mg/mL	NR	
		5–10 kDa, F3	NALKPDNRIESEGG, SSPDIYNPQAGSVT, RQNIGQNSSPDIYNPQAG, VVAEQAGEQGFE HKNKNPF	5%	Maltase: 30% Sucrase: 8%	IC_50_: 0.6 mg/mL	NR	
Soybean	Potential α-glucosidase inhibitor peptides: GSR EAK	Hydrolysate <5 kDa separated by ion exchange chromatography, Fraction C-III isolated and purified by Sephadex G-25, Fraction C-III-2 separated by RP-HPLC, Fraction C-III-2a collected.	GSR EAK	NR	IC_50_: 20.4 μM IC_50_: 520.2 μM α-glucosidase (5NN8): - GSR and EAK bind differently from Acarbose; they bind close to active site, mainly through van der Waals contacts, anion-π interactions, and hydrogen bonds.	NR	NR	[65]
Soybean	Most frequently occurring peptide in soybean proteins with DPP-IV inhibitor activity: GA, GP, and PG	NR	GA, GP, PG	NR	NR	Most frequently occurring peptides in soybean proteins having DPP-IV inhibitor activity	NR	[123]
Soy and Lupin	Potential DPP-IV inhibitor peptides: Soy 1: IAVPTGVA Lup 1: LTFPGSAED	NA (Peptides were synthesized)	Soy 1: IAVPTGVA Lup 1: LTFPGSAED	NR	NR	IC_50_ 106 μM 228 μM DPP-IV (4PNZ): Soy 1 N-terminus and C-terminus matched the binding of omarigliptin. Other interactions include salt bridge, ionic network, π-π stacking, electrostatic interaction, and ionic interactions.	NR	[126]
Common bean (*Phaseolus vulgaris*)	Potential DPP-IV inhibitor peptides detected in unfractionated protein hydrolysate: LAPPG, KLLLRRLQ, REYLLVAQ, LRENNKLMLLELK, RLLLKLRQ	Unfractionated protein hydrolysate	LAPPG, KLLLRRLQ, REYLLVAQ, LRENNKLMLLELK, RLLLKLRQ	NR	NR	Potential activity predicted by BIOPEP database.	NR	[59]
Common bean (*Phaseolus vulgaris* L.)	Potential DPP-IV and α-glucosidase inhibitor peptides: KTYGL, KKSSG, CPGNK, and GGGLHK	Unfractionated protein hydrolysate	KTYGL KKSSG CPGNK GGGLHK	NR	% inh/mg 36.30 ± 8.80 49.34 ± 6.50 37.60 ± 6.80 46.10 ± 8.30 α-glucosidase (3AJ7): binds outside the active site, mainly through polar interactions, hydrophobic.interactions, and hydrogen bonds.	IC_50_ (mg/mL) 0.03 ± 0.00 0.64 ± 0.16 0.87 ± 0.02 0.61 ± 0.10 Diprotin A: 0.02 ± 0.00 DPP-IV (1RWQ): bind to catalytic site, mainly through hydrogen, hydrophobic, polar, and cation π bonds.	NR	[53]
Black bean (Black-Otomi)	Potential GLUT2 and SGLT1 inhibitor peptides: AKSPLF, ATNPLF, FEELN, and LSKSVL	Unfractionated protein hydrolysate	AKSPLF, ATNPLF, FEELN, LSKSVL	NR	NR	NR	GLUT2 (P12336): binds outside catalytic site, mainly through hydrophobic, polar, cation-π, π-π interactions. SGLT1 (3DH4): binds outside catalytic site, mainly through polar and hydrophobic interactions.	[124]
Black bean	Potential SGLT1, GLUT2, PKC, AMPK inhibitor peptides: AKSPLF, ATNPLF, FEELN, and LSVSVL	Unfractionated protein hydrolysate	AKSPLF, ATNPLF, FEELN, LSVSVL	NR	NR	NR	GLUT2 (P12336): most potent FEELN. SGLT1 (2XQ2): most potent ATNPLF. PKC (4RA5): most potent ATNPLF, binds to catalytic site through hydrogen bond. AMPK (4QFG): most potent ATNPLF, binds to catalytic site through hydrogen bond.	[122]
Common bean (*Phaseolus vulgaris* L.)	Potential DPP-IV inhibitor peptides: SGAM, DSSG, LLAH, YVAT, EPTE and KPKL	Unfractionated protein hydrolysate	SGAM, DSSG, LLAH, YVAT, EPTE, KPKL	NR	NR	Potential activity predicted by BIOPEP database	NR	[38]
Black bean (*Phaseolus vulgaris* L.)	Potential α-amylase, α-glucosidase, DPP-IV inhibitor peptides: AKSPLF, QTPF, FEELN, LSKSVL, and EGLELLLLLLAG	Unfractionated protein hydrolysate	AKSPLF, QTPF, FEELN, LSKSVL, EGLELLLLLLAG	α-amylase (1B2Y): Good potential AKSPLF, FEELN, QTPF, LSKSVL, interacted with the catalytic site (TYR151, HIS201, ILE235), mainly through hydrophobic interactions, polar interactions, and hydrogen bonds.	α-glucosidase (3AJ7): Good potential AKSPLF, FEELN, QTPF, and LSKSVL, interacted with the catalytic site (ASP34, THR83, and ASN32), mainly through hydrogen bonds and polar interactions; only one hydrophobic interaction.	DPP-IV (3W2T): Highest inhibition potential EGLELLLLLLAG, AKSPLF, FEELN, interacted with the catalytic site (ASP192, GLU191, ARG253), mainly through hydrogen bonds, electrostatic or polar interactions, and hydrophobic interactions.	NR	[61]
Pinto bean (*Phaseolus vulgaris* cv. Pinto)	Potential α-amylase inhibitor peptides: PPHMLP, PPMHLP, PLPWGAGF, GDAACCGLPLLP, PPHMGGP, PLPPHDLL, and FNPFPSPHTP	<3 kDa	PPHMLP, PPMHLP, PLPWGAGF, GDAACCGLPLLP, PPHMGGP, PLPPHDLL, FNPFPSPHTP	Peptide sequence detected in protein hydrolysate fraction that has the highest α-amylase inhibitor activity.	NR	NR	NR	[42]
Pinto bean (*Phaseolus vulgaris* cv. Pinto)	Novel potential dual functional (α-amylase and angiotensin converting enzyme) inhibitory peptides: PBp1: PPHMLP PBp2: PLPWGAGF PBp3: PPHMGGP PBp4: PLPLHMLP PBp5: LSSLEMGSLGALFVCM	NA (Peptides were synthesized)	PBp1: PPHMLP PBp2: PLPWGAGF PBp3: PPHMGGP PBp4: PLPLHMLP PBp5: LSSLEMGSLGALFVCM	IC_50_ (mM) 23.33 ± 0.15 15.73 ± 0.06 19.83 ± 0.12 15.80 ± 0.17 10.03 ± 0.47	NR	NR	NR	[62]
Hard-to-cook bean (*Phaseolus vulgaris* L.)	Potential α-amylase and DPP-IV inhibitor peptides: FFL, LLSL, QQEG, and NEGEAH	Unfractionated hydrolysate and <1 kDa	FFL, LLSL, QQEG, NEGEAH	α-amylase (1HNY): potential peptides FFL and NEGEAH interacted with the active site (ASP197, GLU233, and ASP300), mainly through van der Waals contacts, hydrogen bonds, electrostatic, charged, and π interactions.	NR	DPP-IV (1X70): Potential peptides LLSL and QQEG, interacted with S1 and S2 pockets of three pockets of active site, mainly through van der Waals contacts, hydrogen bonds, electrostatic, charged, and π interactions.	NR	[25]
Cowpea bean (*Vigna unguiculata*)	Potential DPP-IV inhibitors: KVSVVAL and TTAGLLE	Unfractionated protein hydrolysate	KVSVVAL, TTAGLLE	NR	NR	DPP-IV (1X70): most potent KVSVVAL, low total energy score and several interactions with catalytic region. TTAGLLE binds with active site at S2 and S3 pockets, mainly through van der Waals and electrostatic interactions.	NR	[57]
Common bean (*Phaseolus vulgaris*)	Potential DPP-IV inhibitor peptide: RGPLVNPDPKPFL	Unfractionated protein hydrolysate	RGPLVNPDPKPFL	NR	NR	DPP-IV (1X70): Dock around S3 pocket, mainly through van der Waals and electrostatic interactions.	NR	[55]

NR = Not Reported; NA = Not Applicable.

## Data Availability

Not applicable.
